# Task-Oriented Biomedical Microrobots: Access, Working Environment, Actuation, Body Design, Detection and Control

**DOI:** 10.3390/mi17091048

**Published:** 2026-09-02

**Authors:** Haoyi Sun, Aiwu Zhou, Tingxi Liu, Zhengnan Sun, Liang Zhou, Ji Hu, Ciprian Iliescu, Xiaosheng Zhang, Yi Zhang

**Affiliations:** 1 School of Integrated Circuit Science and Engineering, University of Electronic Science and Technology of China, Chengdu 611731, China; 202422311026@std.uestc.edu.cn (H.S.); zaw@uestc.edu.cn (A.Z.); liutingxi2003@163.com (T.L.); sunzn@std.uestc.edu.cn (Z.S.); 202422311022@std.uestc.edu.cn (L.Z.); hjqwe0314@163.com (J.H.); 2 National Research and Development Institute in Microtechnologies—IMT Bucharest, 077190 Bucharest, Romania; 3 eBio-Hub Centre of Excellence in Bioengineering, National University of Science and Technology POLITEHNICA Bucharest, 060042 Bucharest, Romania; 4 Academy of Romanian Scientists, 050044 Bucharest, Romania

**Keywords:** biomedical microrobots, task-oriented design, working environment, actuation, body design, detection, control, targeted therapy, closed-loop microrobotics

## Abstract

Biomedical microrobots are often classified by propulsion, materials or fabrication, but clinical translation depends on whether a device can complete a task in a specific biological setting. This review frames biomedical microrobots as task-oriented execution systems. Access and the working environment define the first constraints; actuation and body design convert inputs into useful local work; detection and control close the loop by linking measured states to the next action. Examples from vascular, gastrointestinal, pulmonary, luminal, cellular and biofilm-facing systems show that route, medium, motion, retention, payload function, imaging and post-task fate are coupled rather than separate design labels. Magnetic fields can resist flow or guide motion in confined anatomy, acoustic and optical inputs supply penetrative or local energy, and chemical or biological motors use cues from the surrounding medium. Body design then determines whether movement remains compatible with contact, release, sensing, retrieval and biocompatibility. By focusing on task execution rather than platform identity, this review clarifies what should be preserved in test models, what should be measured, and how fair comparisons can be made before translation.

## 1. Introduction

Biomedical microrobots are untethered or minimally tethered robotic systems at micro- and nanoscale, built for medically relevant movement, manipulation, sensing, delivery or assembly. The field draws on micro/nanofabrication and soft materials, with active matter, biomedical engineering and wireless actuation shaping how systems are powered; energy may come from local fuels or from external magnetic, acoustic, optical and electrical inputs [1]. Their medical appeal lies in size. Small robotic bodies may enter confined anatomical spaces and interact locally with tissues or cells. This supports targeted therapy alongside sampling, diagnosis and minimally invasive manipulation. The same size, however, limits onboard sensing and computation and makes direct visual control difficult [2].

The literature already offers strong technology-centered reviews, but those maps do not fully explain why a system matters for a given biomedical task. Complex biological settings are a central barrier. Blood and mucus are obvious examples. Model fluids that reproduce vitreous rheology and direct tissue contact extend this problem beyond blood and mucus; both can alter propulsion, imaging and control [3]. Imaging reviews make a related point: in vivo visibility must be evaluated by tissue penetration depth, contrast, spatial resolution and clinical compatibility, rather than by optical microscopy alone [4]. Control and autonomy reviews divide the problem into actuation-feedback loops, localization and planning [5]. Materials-integration reviews show that actuation, imaging and biological interaction may need to be integrated within the same robot body [6]. Manufacturing then decides whether multifunctional geometries can be reproduced and moved beyond a demonstration [7]. Because these dependencies determine whether a biomedical task can be completed, a task-oriented review follows them as a linked sequence. The biomedical use defines access and the working environment. The environment constrains actuation, body design integrates motion with function, and detection with control determines whether the task can be observed and adjusted in a practical workflow [8].

The need for this perspective does not rest on a claim that clinical translation is already mature. Rather, microrobot systems are increasingly judged by whether motion, localization, functional action and post-task handling can work together. A task-level analysis is therefore timely because it exposes the link that prevents an otherwise capable platform from completing a biomedical operation and gives different technologies a common practical endpoint.

Deployment mode also changes the endpoint used to judge success. Diagnostic systems are judged by the reliability of sampling or readout, whereas therapeutic systems must additionally demonstrate delivery, local action and post-task handling. Operator-guided, tethered or catheter-assisted systems can retain clinical advantages when imaging and retrieval are essential; autonomous untethered systems require evidence that localization, control and post-task fate remain adequate without continuous manual guidance. These modes therefore represent different task constraints rather than a single performance scale.

Vascular examples make this point concrete. For blood-vessel tasks, a microrobot should be judged under the conditions it will face, not only by motion in a simple test fluid. Flow and red blood cells shape transport, while vessel-wall contact, clot structure and imaging access determine whether intervention is possible; post-use management remains part of the same task. Active-retention swimmers address residence at a vascular site under flow [9]. tPA-anchored nanorobots instead frame thrombolysis as a catheter-assisted, fluoroscopy-guided, retrievable recanalization procedure [10]. Heparinoid-polymer-brush magnetic nanorobots pair thrombolytic action with blood-facing surface chemistry [11]. Newer magnetic bodies and swarms test upstream locomotion or blood-vessel crossing under flow-related constraints [12,13]. These studies are not interchangeable examples of magnetic motion. Each matches a vascular obstacle with a different design answer.

Other settings impose different design priorities and require the same discipline. In the stomach, acid-powered micromotors use gastric chemistry for propulsion and drug delivery against infection [14]. In gastrointestinal disease models, oral microrobot formulations for ulcerative colitis and colorectal cancer must survive transit before they can penetrate mucus or diseased tissue and release therapeutic payloads locally [15,16]. Pulmonary delivery shifts access toward inhalation and residence; inhalable biohybrid microrobots and antibiotic-delivering microrobots for acute bacterial pneumonia illustrate the requirement [17,18]. A related lung metastasis study uses biohybrid microrobots for local delivery of drug-loaded nanoparticles [19]. Device and biofilm tasks make local access plus surface contact more important than long-distance navigation. Endoscope-assisted tympanostomy tube biofilm eradication and liquid-bodied robots for complex surface features illustrate this shift [20,21]. Titanium-mesh biofilm removal adds a different device-facing surface and bactericidal requirement [22]. Cell-facing systems place viability and positioning ahead of raw motion, with cell loading built into the design problem [23,24]. CAR T and immune cell microrobots bring local activation and antitumor activity into the same cell-contact setting, together with imaging [25,26].

Task orientation changes the organization of the review because a microrobot is useful only when the whole task can be completed. The sections follow an analytical dependency rather than a universal chronological order: access and working environment define the conditions, actuation produces work within them, the resulting body must preserve motion and function, and detection/control provides the feedback needed to observe and adjust each step. Detection and control therefore operate across the chain, not only after motion and body function.

**Figure 1:**
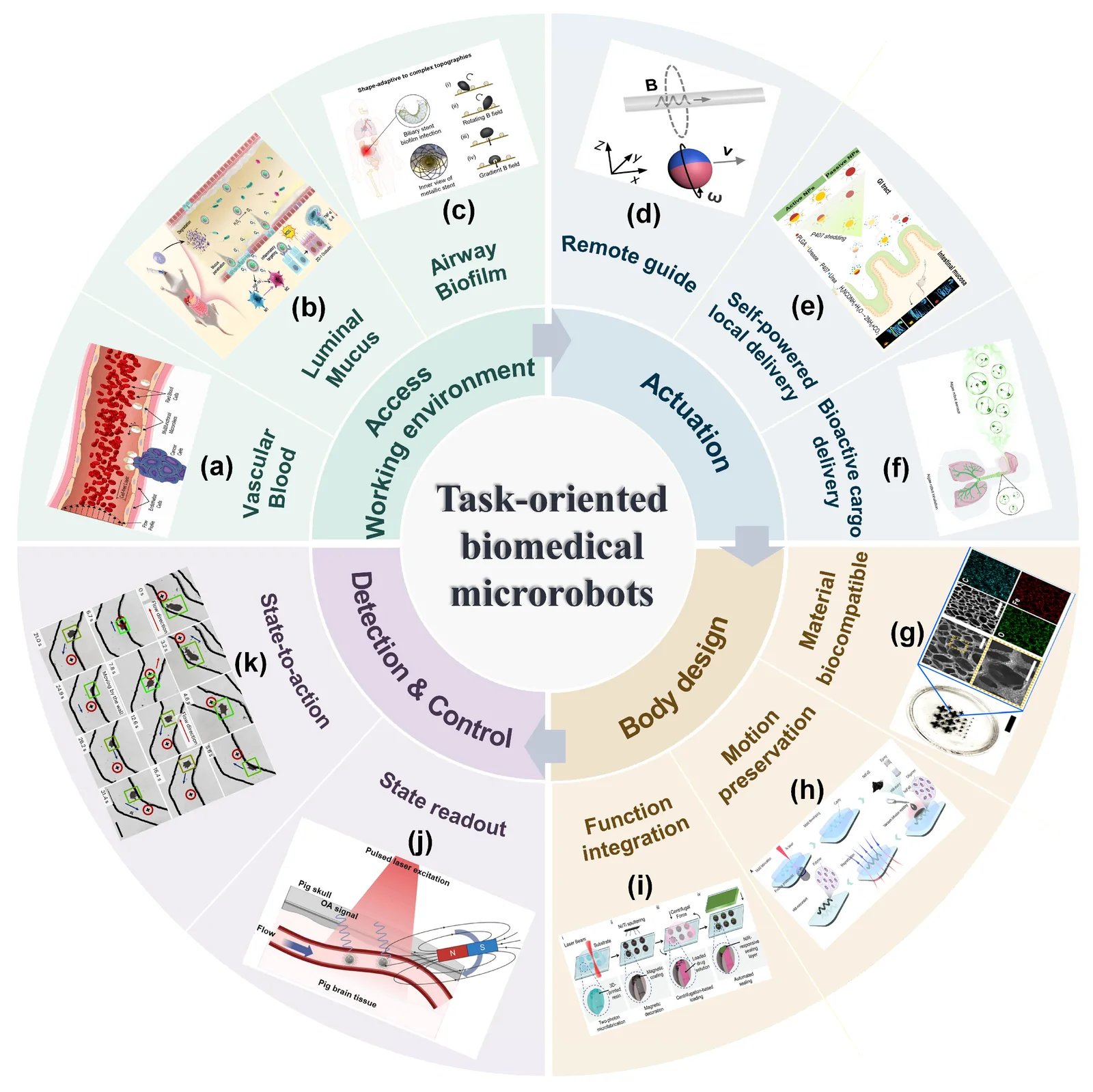
Task-oriented overview of biomedical microrobots across the task chain. Biomedical microrobots are organized here by the task bottlenecks they must solve, rather than by a single platform or technology category. In the access and working environment stage, (**a**) vascular microrollers address cargo transport under blood flow [34], (**b**) chemically propelled designs address inflamed luminal mucus barriers [15], and (**c**) shape-adaptive robots address biofilm-covered luminal or surface topographies [21]. In the actuation stage, (**d**) remote magnetic fields convert external inputs into guided tube navigation [20], (**e**) biodegradable urease-powered nanomotors support gastrointestinal diffusion, distribution and localization [35], and (**f**) living or biohybrid carriers retain motility while delivering bioactive payloads [17]. In the body design stage, (**g**) biocompatible or degradable magnetic scaffolds support task-compatible materials [36], (**h**) fabrication preserves morphology and actuation capacity [12], and (**i**) integrated structures combine loading, protection and triggered release [37]. In the detection-control stage, (**j**) optoacoustic imaging turns microrobot position into a readable state [38], and (**k**) image-derived state is mapped to the next actuation command for closed-loop state-to-action control [39]. Image credits: panel (**a**) was reproduced from Alapan et al., Science Robotics, 10.1126/scirobotics.aba5726 (2020), AAAS, with permission (Ref. [34]); panel (**b**) was reproduced from Luo et al., Science Advances, 10.1126/sciadv.ado6798 (2024), AAAS, with permission (Ref. [15]); panel (**c**) was reproduced from Sun et al., Science Advances, 10.1126/sciadv.adt8213 (2025), AAAS, with permission (Ref. [21]); panel (**d**) was reproduced from Dong et al., Science Advances, 10.1126/sciadv.abq8573 (2022), AAAS, with permission (Ref. [20]); panel (**g**) was adapted with permission from Ref. [36]. Copyright 2021 American Chemical Society; panel (**h**) was reproduced from Wu et al., Science Advances, 10.1126/sciadv.adw1272 (2025), AAAS, with permission (Ref. [12]). Panels (**e**), (**f**), (**i**), (**j**) and (**k**) were adapted from Refs. [35], [17], [37], [38] and [39], respectively, under the Creative Commons Attribution 4.0 International (CC BY 4.0) license.

Figure 1 should not be read as a universal ranking of the four design layers. The decisive bottleneck is task dependent: it is the unmet condition whose failure prevents the next step or the intended biomedical endpoint. At the access and working environment layer, maintaining transport and navigation against physiological blood flow is the initial constraint for vascular microrollers (Figure 1a). For orally administered systems, the starting constraint is survival through the gastric environment followed by passage across the inflamed mucus barrier (Figure 1b). For biofilm treatment on complex luminal or device surfaces, conformal access to the topography is required before the biofilm can be disrupted and debris can be removed (Figure 1c). Once these entry conditions are met, the priority shifts to whether actuation can produce useful work there, whether body design can combine motion with payload function, compatibility and post-task fate, and whether detection-control can verify and adjust the procedure. Figure 1 therefore presents a conditional sequence of design priorities rather than a fixed stage ranking.

Access and working environment are best treated as one design constraint. The entry path places the robot in a biological setting; the setting gives practical meaning to motion, retention, imaging, release and recovery. Catheter-delivered vascular systems must work in flowing blood while remaining easy to image and recoverable when needed. Fluoroscopy-guided tPA nanorobots and hard-magnetic swimmers tested under upstream blood-flow conditions illustrate this requirement [10,12]. Oral gastrointestinal systems face transit after dosing; in colitis or colorectal cancer models, mucus penetration, diseased tissue retention and local release become one environmental problem [15,16]. Inhaled systems couple aerosol-compatible access with pulmonary residence and therapeutic delivery in lung infection models [17,18]. Local device procedures invert the problem: endoscopic access improves visibility; surface coverage and biofilm eradication become the core outputs [20,21]. Cell-facing assays narrow the local environment further, because repeatable contact must be balanced with viability and spatial organization [23,24]. Hyaluronan gels, high-shear biological fluids and wet gastric tissue show why movement or contact cannot be judged in simple buffer alone [27,28,29]. Urease-, catalase- and lipase-powered nanomotors extend this logic to mucus and mucosal transport [30,31,32]. Enzyme-powered swarms add another case: viscous biological settings can support functional transport when diffusion enhancement is the task [33].

Here, actuation means input energy becoming useful work in a defined working environment. It is not a detached catalog of inputs. Magnetic actuation becomes valuable when fields must transmit torque or force through tissue, hold a body under flow, or guide it through confined vascular and perivascular spaces [9,12,13]. Acoustic actuation can drive robots in complex fluids [28] and has been incorporated into bioresorbable hydrogel microrobots pairing propulsion with image-guided operation [40]. Optical or photothermal inputs provide local deformation and gait control when light access is plausible, as shown by lattice soft microrobots with multimodal locomotion [41]. Soft magnetic systems add a deformable route, letting the body adapt its gait to the environment [42]. Chemical actuation is strongest when the task itself supplies the useful reaction window, for example gastric acid or urea-rich fluid [14,30]. Catalase and lipase systems make the same point for mucus-facing settings: substrate availability and barrier penetration decide whether propulsion becomes useful work [31,32].

Material, structural and fabrication choices determine how those inputs become task-capable function. The resulting body must preserve motion while integrating payload, sensing or therapeutic functions and meeting biomedical constraints. Assembly-based magnetic microrobots and 3D-printed magnetic micro/nanorobots show how manufacturing choices set body geometry, magnetic response and cargo capacity [43,44]. Zwitterionic 3D-printed microrobots treat protein adsorption and cell adhesion as material-contact problems linked to immune interaction [45]. Metal–organic-framework-based microrobots illustrate how porous bodies can connect cargo loading with degradation and biomedical function [46]. These examples show that cargo loading, degradation and biological contact are coupled to body architecture rather than being independent add-ons. The combined choices determine whether actuation, cargo or sensing function, biological contact and post-task fate can be engineered as one body design problem [6].

Detection and control close the task chain by converting motion and body function into a measured state, then into the next actuation decision. Dual ultrasound and photoacoustic tracking localizes one microrobot system through complementary contrast mechanisms [47]. TriMag microrobots use the magnetic body for actuation, imaging and hyperthermia [48]. Laser speckle contrast imaging provides another feedback route, allowing a delivery microswarm to be followed and steered in real time [49]. Once a robot is observed, control still has to manage uncertainty. Electromagnetic control studies identify force and localization errors as limits on guidance [50], and autonomous field-of-view planning treats loss of visual access as a control problem [51]. Learning-based controllers are most informative when the measured state, action space and task constraint are specified instead of collapsed into generic automation. The examples cover model-based reinforcement learning for ultrasound-driven autonomous microrobots [39], deep reinforcement learning for magnetic swimming [52], and distribution planning for environment-adaptive swarm navigation [53]. At the swarm level, phase-diagram-guided learning supports process control [54], while AI-assisted analysis classifies multimode behaviors [55].

This review takes a task-oriented view of biomedical microrobots and follows the sequence by which a proposed system becomes useful. Section 2 begins with access and working environment: the entry path and local setting decide what deployment, motion, retention, imaging and recovery mean. Section 3 examines how actuation inputs produce useful work under those constraints. Section 4 turns to the body, asking how material, fabrication and structure preserve motion while adding function and managing compatibility, degradation or retrieval. Section 5 considers measurement and feedback, including how the measured state changes the next magnetic, acoustic, optical or electric input. Section 6 applies these layers to task analysis across workflows, from manipulation and mucosal delivery to pulmonary, cancer, infection and vascular/interventional procedures. The closing sections return to translational priorities: the right comparator, what remains after use, failure modes under realistic conditions and the practical benefit offered by the microrobot.

## 2. Access and Working Environment in Biomedical Tasks

Access sets the first boundary for a biomedical microrobot, but the working environment decides whether the task is physically and biologically plausible. In vessels, the problem is not generic injection but flow, blood-cell effects and clot engagement [10]. Oral entry puts the system into gastric chemistry and mucus before it reaches diseased mucosa [14]. After aerosol delivery, an inhaled microrobot must be judged by deposition and residence, then by activity in the lung [17]. Model systems are useful only when they preserve a key physical feature needed to test a biomedical operating constraint, such as polymer network transport or confinement [27]. Accordingly, this section examines access together with environment-defined tasks: vascular blood settings, oral and luminal barriers, airway or wet surface retention, and model systems that preserve a key physical feature.

### 2.1. Access as the First Boundary of Biomedical Microrobot Tasks

Access should be read as the first operational condition of a task, not as a synonym for administration. Catheter-delivered tPA nanorobots tie access to submillimeter arterial deployment and magnetic actuation; fluoroscopic guidance and post-treatment handling are part of the same procedure [10]. Mg-based micromotors for stomach infection are judged differently because oral dosing places the system directly in gastric acid, with propulsion and antibiotic release occurring together [14]. Inhalable biohybrid microrobots make pulmonary deposition and residence the access problem. Their delivery is judged by activity after aerosol entry rather than by swimming in an open liquid chamber [17]. Endoscope-assisted helical micromachines for tympanostomy tube biofilms introduce an access condition defined by clinical visualization and a narrow device lumen before actuation is considered [20]. Controlled ex vivo or in vitro workspaces also act as access boundaries when the main issue is gentle contact and positional repeatability without loss of cell viability, as in soft microrobots for single-cell transport and spheroid assembly [24].

These examples explain why access is a starting point rather than a complete classification. The entry mode tells the reader where the robot begins. The working environment determines how later claims about motion, visibility, release, contact and recovery should be interpreted. The rest of this section therefore follows the environmental constraint created by access instead of listing delivery modes.

### 2.2. Blood-Related Working Environments After Vascular Entry: Flow, Cellularity and Thrombi

**Figure 2:**
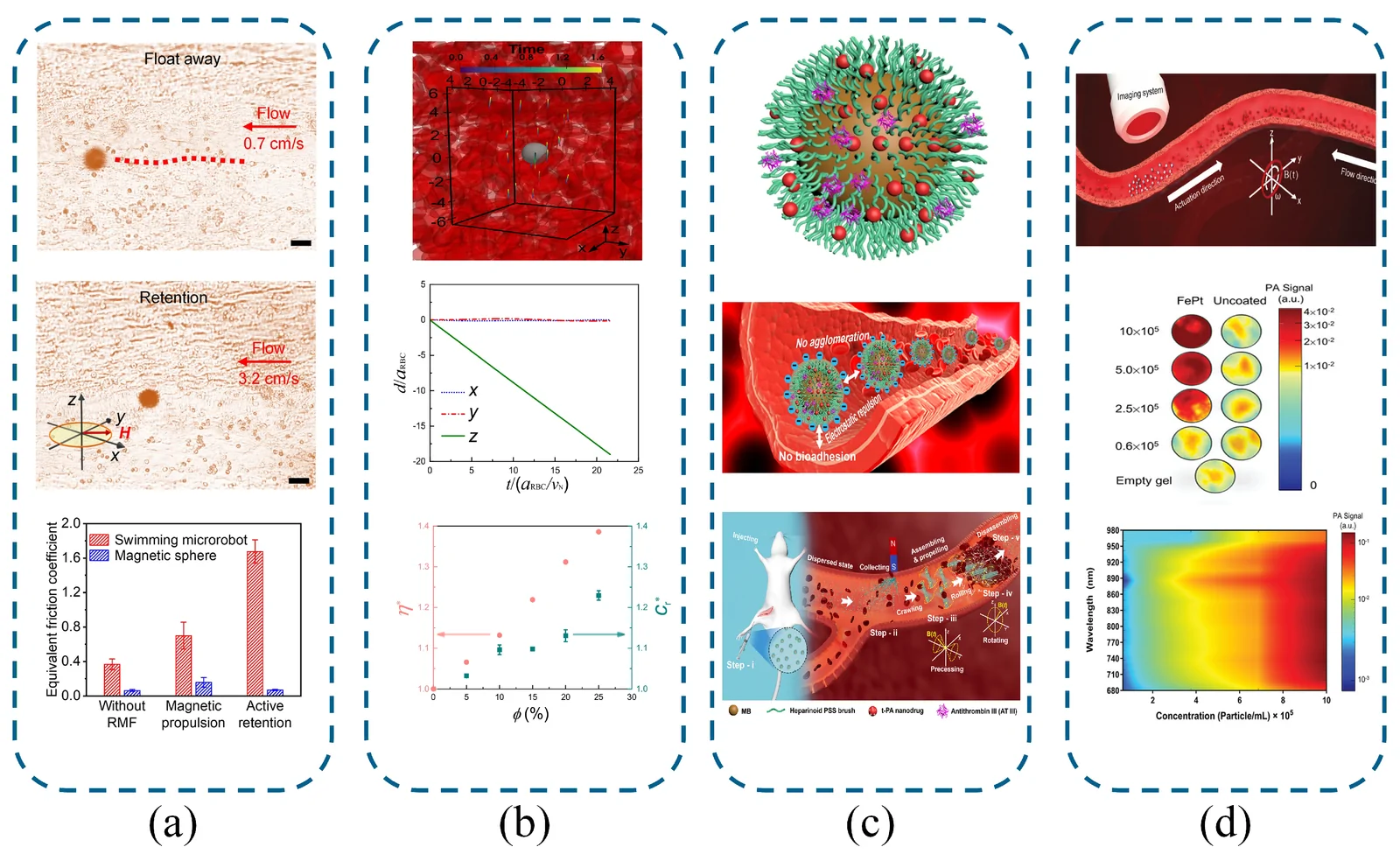
Blood-related working environments after vascular entry. Vascular access becomes useful only when microrobots can address the coupled task problems of flow washout, cellular blood mechanics, thrombus intervention and intravascular visibility. (**a**) Flow-facing retention uses claw-engaged and biolubricated swimming microrobots to resist washout and remain near the vessel wall under blood flow [9]. (**b**) Red-blood-cell suspension models show that cellular blood changes drag, displacement and trajectory, so blood cannot be reduced to a homogeneous viscous fluid [58]. The asterisked symbols are normalized quantities rather than significance markers: η* = η/μ is the relative apparent viscosity of the pure red-blood-cell suspension (μ is the viscosity of the suspending medium), and C_r* = C_r/(6π) = v_N/v_z is the relative resistance coefficient of the microrobot (**c**) Heparinoid-polymer-brush magnetic nanorobots combine magnetic transport with tPA loading and antithrombin III-mediated surface chemistry to treat thrombi as both mechanical and biochemical targets [11]. (**d**) Imageable endovascular microrollers add photoacoustic visibility to vascular delivery, indicating that intravascular task execution may also require a readable state [56]. Image credits: panel (**a**) was reproduced from Li et al., Science Advances, 10.1126/sciadv.adg4501 (2023), AAAS, with permission (Ref. [9]); panel (**c**) was reproduced from Yang et al., Science Advances, 10.1126/sciadv.adk7251 (2023), AAAS, with permission (Ref. [11]). Panels (**b**) and (**d**) were adapted from Refs. [58] and [56], respectively, under the Creative Commons Attribution 4.0 International (CC BY 4.0) license.

One blood-related environment is moving blood near vessel walls, with transport competing against washout. Surface microrollers designed for physiological blood flow showed the need for cargo loading to survive in a moving fluid, not only for motion [34]. Claw-engaged and biolubricated microrobots approached the same problem through body shape and surface lubrication, creating active retention in blood vessels [9] (Figure 2a). FePt surface microrollers connected magnetic rolling with endovascular imaging, so visibility became part of the flow-facing design problem [56] (Figure 2d). A femtosecond laser printing route produced hard-magnetic swimmers evaluated by their ability to move against slow blood-like flow, making motion against flow a vascular criterion [12]. Self-adhesive microgel swarms for aneurysm embolization make local stopping and sealing the intended vascular outcome rather than a transport failure [57].

Beyond flow washout, vascular bifurcations introduce a branch selection problem. Alves et al. modeled spherical microrobots 50–1000 µm in diameter in idealized cerebral-artery bifurcations at maximum blood velocities of 0.25–0.65 m s^−1^, and mapped the magnetic field gradients required for branch guidance as functions of robot size, artery geometry, initial position and intermediate target placement [59]. Because the vessels were represented as straight and smooth, the flow was steady and non-pulsatile, and the route was specified through intermediate targets, this study supports scale-aware waypoint steering rather than autonomous closed-loop path planning in a tortuous microvascular network. Experimental systems address complementary constraints: clawed bodies improve wall-facing retention [9], whereas hard-magnetic helical geometries support upstream propulsion in whole blood in vitro [12]. Hematocrit and robot-to-cell scale alter resistance [58], while photoacoustic visible FePt microrollers show how propulsion can remain observable under tube flow [56]. None of these studies tested active shape deformation during bifurcation transit, so its value for navigating tortuous microvessels remains to be established.

Another blood-related environment is cellular blood. Unlike a simple flowing liquid, it contains deformable cells acting as mechanical and biological participants. Simulations of a microrobot propelled through blood linked hematocrit and robot geometry to drag, with size and cell deformability contributing to the same resistance [58] (Figure 2b). A study of surface-active microrobots also linked local surface flows to nearby red-blood-cell reorganization and to motion unlike an inert body in the same suspension [60]. The claw-engaged vascular robot uses red-blood-cell membrane camouflage, making red blood cells part of the biological surface design rather than only obstacles to motion [9]. This result addresses active retention under the tested flow conditions; it does not by itself establish long-term immune compatibility, clot penetration or post-task retrieval. Neutrophil-based biomotor engineering extends this point by turning living blood cells into active carriers for thrombolytic nanodrug delivery [61].

A further blood-related environment is the thrombus. Here the vessel contains a pathological solid that must be transformed, lysed or removed. tPA-anchored Fe_3_O_4_@mSiO_2_ nanorobots combined catheter access with magnetic steering and fluoroscopic monitoring to recanalize arterial segments in vivo [10]. Heparinoid-polymer-brush magnetic nanorobots treated the clot as both a mechanical obstacle and a biochemical target, using swarming and tPA-mediated lysis [11] (Figure 2c). Remote delamination and magnetization of clot debris shifted the outcome from passive dissolution toward active removal of clot material [62]. A magnetically actuated self-clearing catheter is not a microrobot, but it sets a procedural benchmark that small-scale clot-removal systems will eventually have to face [63]. Engineered microrobots carrying bacterial outer-membrane vesicles add an immunotherapeutic form of thrombus treatment, where the target is a living pathological structure as well as a physical blockage [64].

Taken together, these cases indicate a task-dependent vascular criterion rather than a universal ranking. A blood-facing microrobot has to remain useful under flow and around cells, then engage the vessel or clot, remain visible enough for intervention and manage residual material after the task. Speed matters only when it supports one of these vascular functions.

### 2.3. Gastric Fluids, Mucus and Inflamed Mucosa After Oral or Luminal Entry

**Figure 3:**
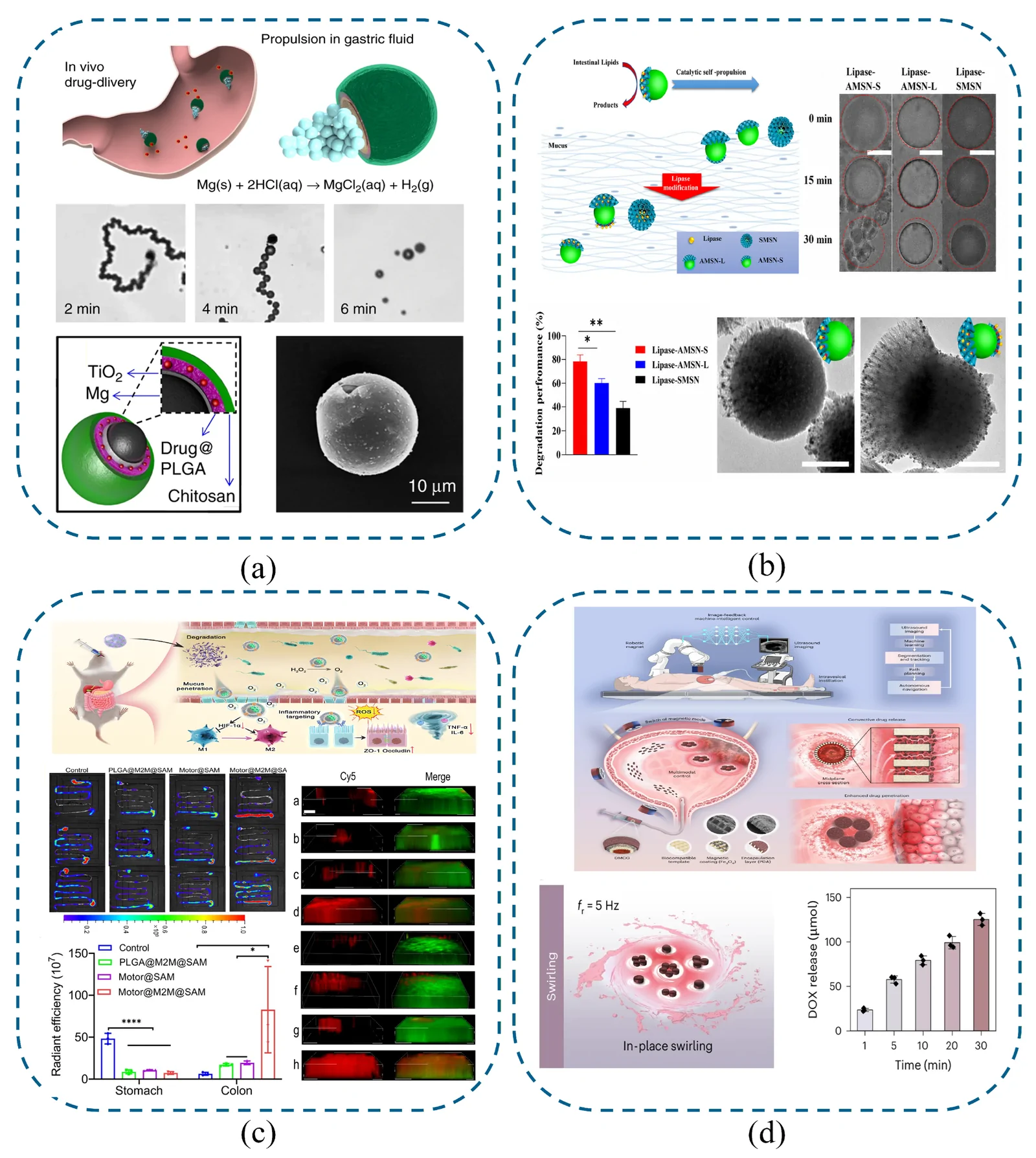
Oral and luminal working environments. After oral or luminal entry, the solution must be tailored to gastric chemistry, mucus barriers, inflamed mucosa and local antimicrobial availability; the solution is not generic propulsion, but active transport matched to the luminal microenvironment. (**a**) Mg-based gastric micromotors use gastric acid as a local fuel window for propulsion while layered TiO_2_/PLGA/chitosan structures carry antibiotic payloads [14]. (**b**) Lipase-powered asymmetric silica nanomotors couple lipase-mediated lipid degradation with a tailored head–tail structure to enhance mucus penetration [32]. (**c**) Macrophage-mimetic Janus motors exploit ROS-rich inflamed mucosa for propulsion, mucus penetration, inflammatory targeting and local retention in ulcerative colitis models [15]. (**d**) Machine-intelligent multimodal algebots combine magnetic actuation, imaging/robotic guidance and local drug transport for intracavitary chemotherapy [70]. Image credits: panel (**c**) was reproduced from Luo et al., Science Advances, 10.1126/sciadv.ado6798 (2024), AAAS, with permission (Ref. [15]). Panels (**a**), (**b**) and (**d**) were adapted from Refs. [14], [32] and [70], respectively, under the Creative Commons Attribution 4.0 International (CC BY 4.0) license.

Oral and luminal entry first exposes a microrobot to chemistry that may power the task, disable the body, or become the readout. Artificial micromotors in the mouse stomach established that synthetic motors can operate in gastric fluid in vivo, with acid forming part of the operating window [65]. Mg-based micromotors for *Helicobacter pylori* treatment used the same acidic gastric setting to drive propulsion and local antibiotic release [14] (Figure 3a). A gastric-fluid-gradient study showed that pH and conductivity can switch the device between chemical motion, magnetic rolling and electrical operation [66]. Urease-powered oral micromotors make enzymatic activity the driving logic for stomach delivery rather than relying on acid consumption alone [67]. Pollen-derived micromotors for gastric ulcer treatment show how naturally structured particles can be adapted to a diseased gastric setting [68]. Magnetic microrobots with fluorescent internal memory make gastric acidity a sensing output, not only a condition to survive [69].

Mucus turns the same oral or luminal entry into a barrier crossing problem. Hyaluronic-acid-based nanomotors were designed around mucosal-barrier penetration for antimicrobial treatment, making the barrier the central design constraint [71]. RoboCap is larger than the microrobot systems emphasized here, but it shows how strongly gastrointestinal delivery can depend on physically clearing the mucus layer [72]. Lipase-powered asymmetric silica nanomotors used head–tail asymmetry with lipase-mediated lipid degradation to recast mucus crossing as an enzyme–substrate transport problem. This improved penetration without catalase, peroxide decomposition or oxygen bubbles [32] (Figure 3b). Catalase-powered nanobots were evaluated for overcoming the mucus barrier, linking single-particle motion to transport through a biologically relevant obstacle [31]. Machine-intelligent multimodal algebots show how luminal or intracavitary access can become guided local chemotherapy. Magnetic transport, imaging feedback and local drug release operate as one task chain [70] (Figure 3d). In bladder cancer sonodynamic therapy, urease-driven nanomotors apply the same barrier-crossing principle by improving penetration through mucosa and tumor tissue [30].

Direct comparison is possible only within the model and endpoint reported by each study. In an HT29-MTX mucus-secreting model, where a 15 μm three-dimensional crop represented the mucus-occupied volume, catalase nanobots driven by 200 mM H_2_O_2_ achieved 28% crossing in 15 min, 60 times the passive particle result [31]. In a separate transwell model containing an approximately 3 mm layer of 3 wt% mucin with triacetin, lipase modification increased cumulative transport of the short-tail asymmetric silica nanomotor from approximately 7% to approximately 80%, while its propulsion velocity was more than 10 times that of the unmodified particle [32]. Hyaluronic-acid/urease nanomotors were tested across 100 μL of 2 wt% mucin with 100 mM urea. This study reported a functional endpoint rather than penetration distance: at 24 h, chloramphenicol-loaded nanomotors produced an 11.6 ± 2.6-fold greater reduction in bacterial proliferation than free chloramphenicol [71].

At the capsule scale, RoboCap used 200–800 μm studs to clear a reported 500–800 μm intestinal mucus layer during approximately 35 min of operation. It required approximately 250 mW and increased vancomycin permeability by more than 10-fold ex vivo and more than 20-fold in vivo [72]. These data do not support a universal optimum based on nominal mucus thickness alone. Selection must also match mucus composition, device scale, propulsion source and the task endpoint, which may be particle crossing, local mucus removal or drug uptake. Only RoboCap reports electrical power; the enzyme-powered studies instead specify substrate concentrations, so their energy efficiency cannot be placed on one numerical scale.

Inflamed mucosa adds disease chemistry and immune behavior to the barrier problem. A biomimetic sweeping microrobot for ulcerative colitis was designed to remove pathogenic cell-free DNA and reactive oxygen species while operating in colonic mucus [73]. Macrophage-mimetic Janus nanomotor microspheres for ulcerative colitis combine oral protection with inflammatory targeting and local motion in the diseased intestine [15] (Figure 3c). Twin-bioengine self-adaptive micro/nanorobots use enzyme actuation followed by macrophage relay, linking propulsion to immune cell behavior in the same gastrointestinal inflammation task [74]. Magnetic living hydrogels for intestinal localization and diagnosis show that local retention can matter as much as movement through the lumen [75]. Enteric micromotors that selectively position and spontaneously propel in the gastrointestinal tract further show that local positioning is a biological constraint, not a generic delivery label [76].

This subsection is therefore not a single gastrointestinal story. Gastric acid sets the chemical window for propulsion or sensing. Mucus sets the transport barrier. Inflamed mucosa adds disease-specific chemistry and immune contact. Access links these cases because the systems enter through oral or luminal spaces, but the design rules come from the environment reached after entry. The next actuation chapter should therefore ask how an input works in acid, mucus or inflamed tissue before comparing actuation modes in the abstract.

### 2.4. Airway Deposition, Wet Tissue Contact and Biofilm Surfaces

**Figure 4:**
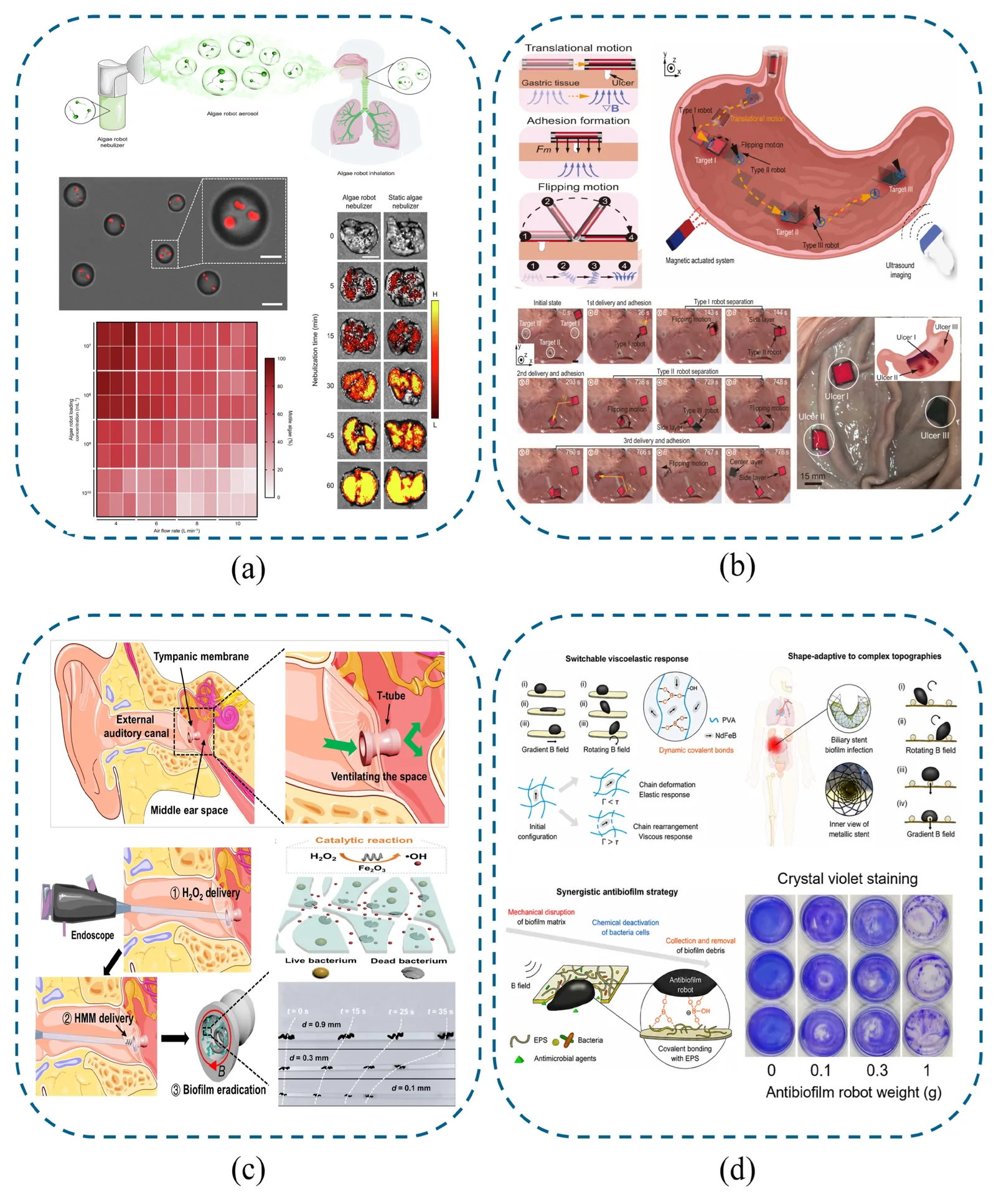
Deposition, wet contact and biofilm surfaces. When the task shifts from entering a body space to remaining and acting on a surface, success depends on deposition, adhesion, confined access, surface adaptation and biofilm removal. (**a**) Inhalable algae biohybrid microrobots show that pulmonary access requires aerosol deposition together with retained motility after nebulization [17]. (**b**) Magnetic multilayer soft robots convert field-driven motion into on-demand adhesion, maintenance and detachment on wet gastric tissue [29]. (**c**) Endoscope-assisted helical micromachines address device lumen biofilms by coupling clinical access, local H_2_O_2_ delivery and magnetic mechanical disruption [20]. (**d**) Liquid-bodied antibiofilm robots use switchable viscoelasticity to deform across complex topographies, disrupt biofilm matrices, chemically deactivate bacteria and collect debris [21]. Image credits: panel (**c**) was reproduced from Dong et al., Science Advances, 10.1126/sciadv.abq8573 (2022), AAAS, with permission (Ref. [20]); panel (**d**) was reproduced from Sun et al., Science Advances, 10.1126/sciadv.adt8213 (2025), AAAS, with permission (Ref. [21]). Panels (**a**) and (**b**) were adapted from Refs. [17] and [29], respectively, under the Creative Commons Attribution 4.0 International (CC BY 4.0) license; parts of the original source panel for panel (**b**) were created with BioRender.com.

Airway tasks are organized by deposition and residence before retained activity, not by injection. Inhalable biohybrid microrobots were built around aerosol delivery and activity in the lung; non-invasive entry mattered because the microrobots remained functional after nebulization [17] (Figure 4a). Nanoparticle-modified algae microrobots for acute bacterial pneumonia paired biological swimming with antibiotic-loaded nanoparticles in an infected lung setting [18]. Biohybrid microrobots for lung metastasis extended the pulmonary task from infection to local active delivery of drug-loaded nanoparticles for cancer intervention [19]. In bronchial delivery, active microgel swarms make the airway tree part of the operating environment because steering and deposition occur within branching passages [77].

Wet luminal tissues shift evaluation from travel to maintained contact. A magnetic multilayer soft robot for on-demand targeted adhesion was evaluated on wet gastric tissue, where attachment and detachment were the central outputs [29] (Figure 4b). A 3D-printed hydrogel design inspired by pollen grains uses body architecture as a switchable contact module: magnetic rolling brings the body forward, anchoring holds it, and release completes the local action [78]. Self-adhesive microgel swarms in aneurysm embolization give a vascular version of the same contact logic, because a local adhesive state creates the treatment effect [57]. Magnetic living hydrogels in the intestine show how retention within a luminal setting can support diagnosis as well as delivery [75].

Biofilm-bearing surfaces define another environment because the target is a structured community attached to a device or tissue surface. Endoscope-assisted magnetic helical micromachines for tympanostomy tube biofilms linked endoscopic visibility to confined delivery; eradication was measured at the local biofilm target [20] (Figure 4c). Liquid-bodied antibiofilm robots with switchable viscoelastic response were designed for complex surface features, where deformability and surface contact determine cleaning performance [21] (Figure 4d). Swarming magnetic photoactive microrobots for dental implant biofilms show that implant-associated surfaces require local access to be coupled with antimicrobial action [79]. Multimodal magnetic microrobots for titanium mesh biofilm removal add a clinically recognizable implant material to the same surface-cleaning problem [22]. Self-locomotive antimicrobial microrobot swarms demonstrate that active motion can improve biofilm elimination in a broader biomaterials setting [80].

The shared requirement is maintained contact outside an open chamber. A pulmonary robot must deposit and remain active. A wet tissue robot must attach, then release in a controlled way. A biofilm robot must first reach a surface-bound target; disruption and clearance are the task outputs. These requirements connect access back to actuation, because force input is useful only if it creates the required contact state in the actual working environment.

### 2.5. Feature-Preserving Test Environments: Polymer Networks, Viscoelastic Fluids and Confinement

The studies in this subsection are not treated as whole-body environments. They are included because some non-in vivo systems isolate a feature that would otherwise be hard to see inside a complex animal experiment. A polymer network can test whether a helical body is small enough to pass through a mesh. A viscoelastic solution can test whether elastic stress changes swimming. A confined channel can test whether geometry changes the locomotion mode. These systems should be used as tests that preserve key features, not as substitutes for biological validation.

Feature preservation can also be biological. Yuan et al. developed an immunocompetent liver-on-a-chip that incorporated six human cell lines, blood- and bile-side perfusion, and chemotaxis-responsive immune cell migration, and used targeted cellular depletion to resolve cell-type-specific contributions to drug-induced hepatotoxicity [81]. Although the study did not investigate microrobots, the platform captures selected hepatic, immune and flow-related features in a multicellular, perfused system. Such models could complement other preclinical tests of future liver-directed microrobots, particularly for examining local cellular interactions and material- or payload-associated toxicity; however, their cell-line-based design and limited throughput should be considered when assessing translational relevance.

**Figure 5:**
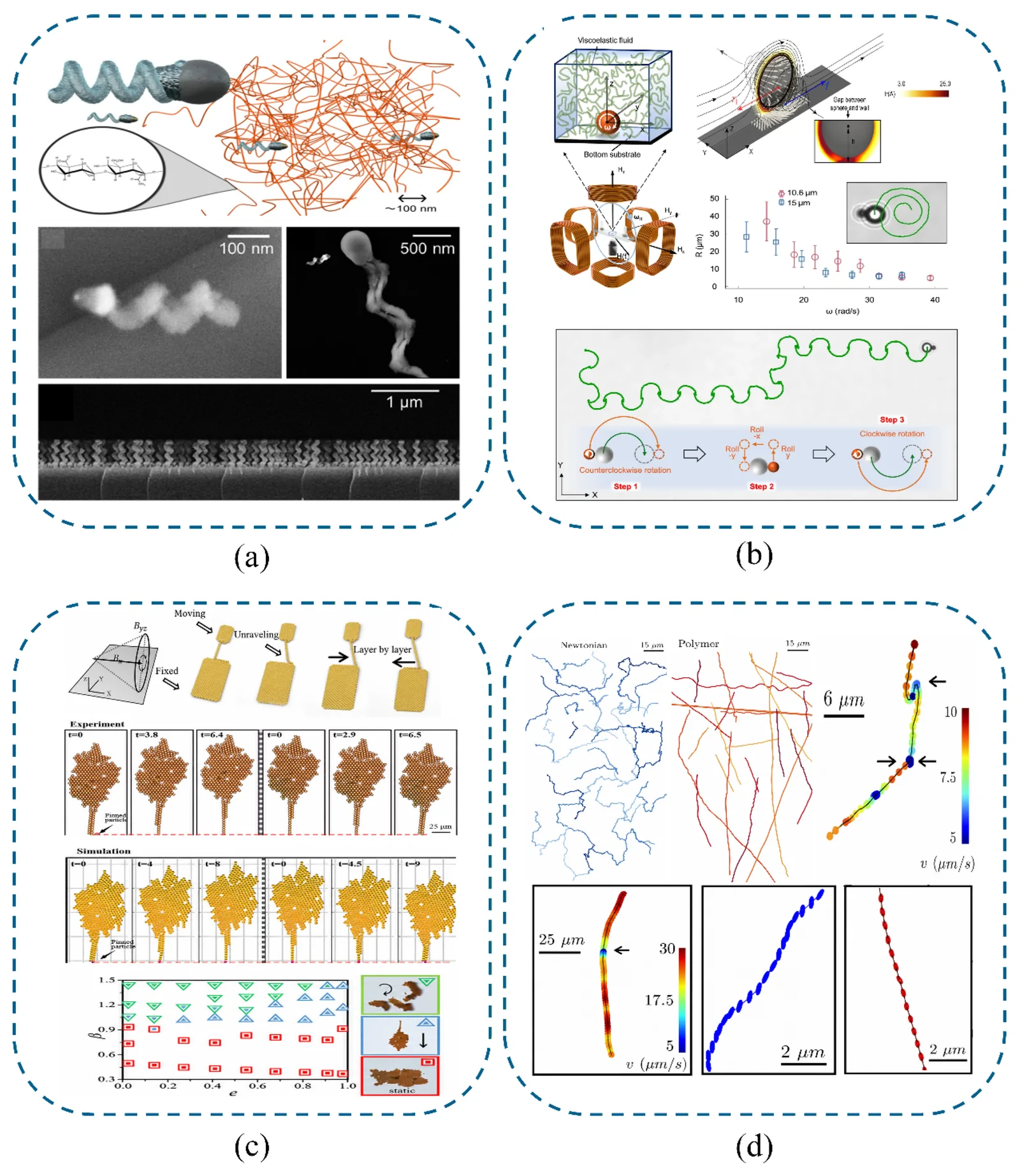
Feature-preserving test environments. Model environments are useful when they preserve the physical constraints that define a task, including polymer mesh size, viscoelastic wall effects, confinement and polymeric-fluid swimmer dynamics. (**a**) Hyaluronan gels and polymer networks retain mesh size constraints that micro- and nanopropellers must overcome in barrier-rich settings [27]. (**b**) Rollers in viscoelastic fluids reveal wall-coupled sliding and trajectory changes that would be missed in simple Newtonian buffers [84]. (**c**) Magnetic swarm unraveling in artificial microtubules shows how confinement can be engineered into directed cargo transport rather than treated only as an obstacle [87]. (**d**) Bacterial swimmer trajectories in polymeric solutions illustrate how complex fluids alter motility; this panel is a model environment constraint, not direct evidence of a synthetic therapeutic microrobot [82]. Image credits: panel (**a**) was adapted with permission from Ref. [27]. Copyright 2014 American Chemical Society. Panel (**c**) was reproduced with permission from Wang et al., Artificial Microtubules from Microrobot Swarm Unraveling for Directed Cargo Transport under Confinement, Advanced Materials, doi: 10.1002/adma.202502621; published by John Wiley & Sons, 2026 (Ref. [87]). © 2025 Wiley-VCH GmbH. Panels (**b**) and (**d**) were adapted from Refs. [84] and [82], respectively, under the Creative Commons Attribution 4.0 International (CC BY 4.0) license.

Polymer networks and gels are useful when mesh size or polymer mechanics controls transport. Magnetic nanopropellers in hyaluronan gels showed how polymer network constraints can distinguish nanoscale propellers from larger micropropellers [27] (Figure 5a). *E. coli* run-and-tumble dynamics in polymeric solutions demonstrate that polymer concentration and viscoelastic response can change trajectories even for biological swimmers [82] (Figure 5d). Hyaluronic-acid nanomotors remain application-facing mucus studies, but their interpretation still depends on whether the test system preserves the mucosal-barrier feature [71]. Catalase-powered nanobots make the same point more directly by measuring disruption and crossing of an intestinal mucus barrier [31].

Viscoelastic fluids show that motion cannot be predicted from viscosity alone. Sperm-driven micromotors in oviduct fluid and viscoelastic formulations showed that a biohybrid body may behave differently in reproductive-fluid-like settings than in buffer [83]. Backward sliding of rollers on surfaces in viscoelastic fluid demonstrates that wall contact and elastic stress can even reverse the intuitive link between rotation and translation [84] (Figure 5b).

Confinement adds geometry to the environmental feature under test. Soft-bodied adaptive microrobots in fluid-filled confined spaces showed that channel geometry and actuation frequency can induce transitions between rolling, crawling, swimming and helical surface motion [85]. Microrobot swarms that actively explore and reconstruct vascular networks treat the network itself as the working environment, not as a passive container [86]. Artificial microtubules formed by microrobot swarm unraveling show that confinement can also be used constructively for directed cargo transport [87] (Figure 5c). Endoscopic biofilm delivery in a tympanostomy tube provides a clinical reminder that confined geometry is not only a laboratory concern [20].

### 2.6. Design Rules Set by Access and Working Environment

Access and working environment set the first design rules for the rest of the review. In vascular tasks, flow resistance and cellular interaction come before any claim about clot engagement, imaging or residual material. In oral and luminal tasks, acid response and mucus penetration have to be read together with local retention, inflammation and disease-specific release. In airway and wet surface tasks, deposition or residence only matters if adhesion, detachment, surface coverage and debris clearance can follow. In systems that preserve key features, the early question is whether the chosen test environment preserves the factor that controls motion or contact.

These distinctions prepare the actuation discussion that follows. Magnetic, acoustic, optical, chemical and biological inputs should not be compared as a detached menu. Each input matters only when it can transmit useful work through the environment created by access. A vessel may require torque, mucus may require propulsion, a wet surface may require force, a bronchial branch may require guided motion, and a confined network may require control of collective transport. The same logic carries forward into materials and fabrication, then into imaging and control. Every later design choice is judged against the environment in which the biomedical task is meant to occur.

## 3. Actuation: Converting Input Energy into Useful Work in Working Environments

Actuation is where an input earns its value: it has to pass through a specific microrobot body and become work. In magnetic microrobots, the input can set orientation and torque while also generating force under six-degree-of-freedom control [88]. The resulting motion is still bounded by localization error and by uncertainty in external forces, even in engineered testbeds [50]. Helical magnetic micromachines illustrate the body term neatly. A rotating field becomes corkscrew swimming only when it meets a matched helical geometry [89]. Bubble-based acoustic microrobots offer a parallel acoustic case, where geometry shapes acoustic streaming into distinct locomotion states [90]. Thus the actuation question is not the label attached to the energy source. It is whether input and body can use the surroundings to create the local action required by the task, from steering or stopping to release, heating, disruption or retrieval. Table 1 therefore compares only directly reported task-relevant metrics and retains the experimental boundary of each representative study.

### 3.1. Magnetic Actuation: Field Control, Torque and Force in Moving or Confined Spaces

**Figure 6:**
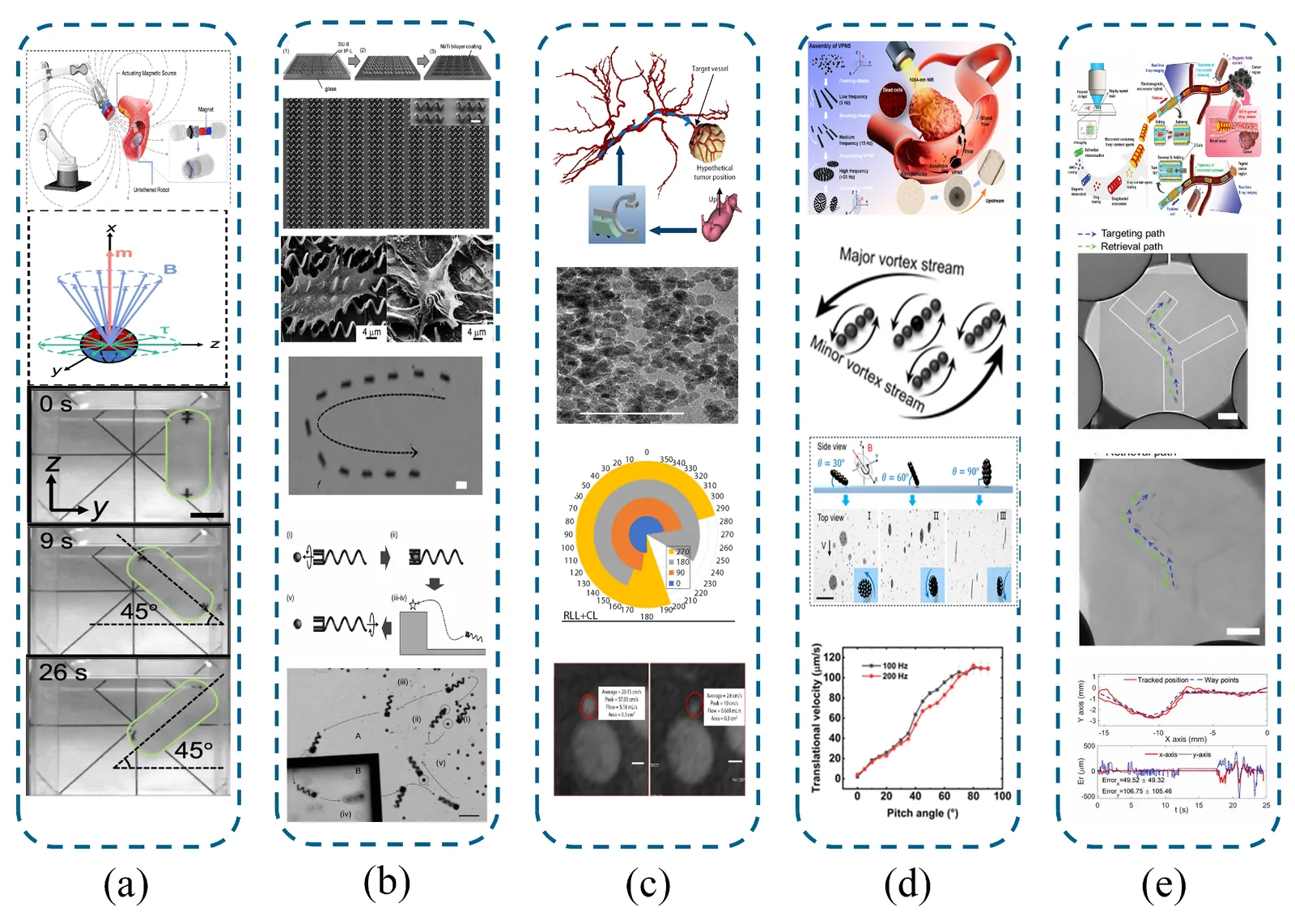
Magnetic actuation as task-directed work. Magnetic input is valuable when field-generated torque or force can be translated into pose control, propulsion, vascular navigation, upstream retention, release or retrieval under task constraints. (**a**) A single anisotropic soft magnet enables three-degrees-of-freedom orientation manipulation, defining magnetic pose control as an orientation task rather than full six-degree position-and-orientation control [96]. (**b**) Rotating magnetic fields drive helical micromachines through corkscrew propulsion, turning and cargo handling [89]. (**c**) Human-scale hepatic-artery navigation studies show that vascular magnetic navigation must account for field gradients, path geometry and body-position compensation [97]. (**d**) Reconfigurable vortex-like magnetic nanoparticle swarms use collective magnetic response to support upstream motility and retention under flow [98]. (**e**) Self-rolled magnetic microrobots couple navigation with imaging visibility, triggered release and retrieval, illustrating a multi-step magnetic task chain rather than a single actuation mode [99]. Image credits: panel (**b**) was reproduced with permission from Tottori et al., Magnetic Helical Micromachines: Fabrication, Controlled Swimming, and Cargo Transport, Advanced Materials, doi: 10.1002/adma.201103818; published by John Wiley & Sons, 2012 (Ref. [89]). © 2012 WILEY-VCH Verlag GmbH & Co. KGaA, Weinheim. Panel (**c**) was reproduced from Li et al., Science Robotics, 10.1126/scirobotics.adh8702 (2024), AAAS, with permission (Ref. [97]). Panel (**e**) was reproduced with permission from Nguyen et al., A Magnetically Guided Self-Rolled Microrobot for Targeted Drug Delivery, Real-Time X-Ray Imaging, and Microrobot Retrieval, Advanced Healthcare Materials, doi: 10.1002/adhm.202001681; published by John Wiley & Sons, 2021 (Ref. [99]). © 2021 Wiley-VCH GmbH. Panels (**a**) and (**d**) were adapted from Refs. [96] and [98], respectively, under the Creative Commons Attribution 4.0 International (CC BY 4.0) license.

Open liquid workspaces give magnetic actuation its simplest control case, before biomedical constraints complicate it. A single-body magnetic microrobot can be commanded in six degrees of freedom; this separates field-imposed orientation and translation from the body design itself [88]. With a single anisotropic soft magnet, an untethered robot achieved three-degrees-of-freedom orientation manipulation. That result distinguishes orientation control from full six-degree position-and-pose control [96] (Figure 6a). Disturbance-aware electromagnetic control studies add a more difficult requirement: the control strategy has to tolerate force uncertainty and localization error, not just ideal field models [50]. Magnetic helical micromachines then convert rotating fields into corkscrew swimming and cargo transport, so waveform and body geometry become inseparable from propulsion [89] (Figure 6b). These mechanism studies set variables that later biomedical examples still have to manage. Field direction and frequency matter, but so do gradient, magnetization pattern, body shape and feedback accuracy.

Vascular flow changes the magnetic problem. The device has to resist washout and gain upstream bias while remaining controllable at human-relevant distances. In human-scale hepatic-artery navigation, magnetic resonance gradients were combined with patient positioning; field strength and gravity became part of endovascular control [97] (Figure 6c). A reconfigurable vortex-like paramagnetic nanoparticle swarm was built for upstream motility. Its value lies in collective magnetic organization for transport against flow, rather than in simple downstream drift [98] (Figure 6d). The hard-magnetic printing strategy makes a related point by tying programmed magnetization to swimming against flow, with controlled motion against a vascular-like stream as the actuation target [12]. In vessels, force margin matters together with upstream bias, wall interaction and imaging feedback. Speed in a still chamber is only a partial test.

Inside confined lumens and tortuous channels, magnetic actuation is shaped by geometry and contact state as much as by what must happen after the task. Endoscopy-assisted navigation of biohybrid soft microrobots showed that magnetic steering can work with rapid deployment and visibility in deep, narrow digestive spaces [100]. Self-rolled magnetic microrobots pushed this workflow further: local drug release and X-ray-visible guidance were linked to catheter-assisted recovery, so navigation, therapy and removal were tested together [99] (Figure 6e). Soft magnetic microrobots with environmental adaptive multimodal locomotion enabled transitions between motion states during targeted delivery, indicating how field conditions and body geometry select the useful gait [42]. In these spaces, the useful work is controlled contact with a channel or tissue wall. Open-volume translation is not enough.

On wet tissue and diseased surfaces, magnetic input becomes useful only through local mechanical work. Contact, adhesion and detachment are part of that work rather than secondary handling steps. A magnetic multilayer soft robot paired magnetic motion with a separable adhesive layer to achieve on-demand targeted adhesion on gastric tissue under ultrasound tracking [29]. In thrombus treatment, remote delamination and magnetization of clot debris moved the task beyond passive dissolution toward magnetically assisted detachment and recovery of clot material [62]. Liquid-bodied antibiofilm robots used field-triggered viscoelastic switching to cross complex surface features and eradicate biofilm [21]. For these targets, travel distance is secondary. The question is whether magnetic work can press into a diseased surface, attach long enough to act, then shear, reorganize or retrieve material.

Across liquid phantoms, blood vessels, confined channels and diseased surfaces, magnetic actuation is most persuasive when remote fields are converted into a specified mechanical action. Magnet size and field strength matter, but they are only part of the design space. Waveform, gradient and frequency define the field program; magnetization, compliance and contact mechanics define the body response. Imaging feedback and post-task recovery then decide whether the same magnetic input looks strong in one task and weak in another.

### 3.2. Acoustic, Light, Electric and Thermal Actuation for Penetrative Energy Delivery and Local Work

**Figure 7:**
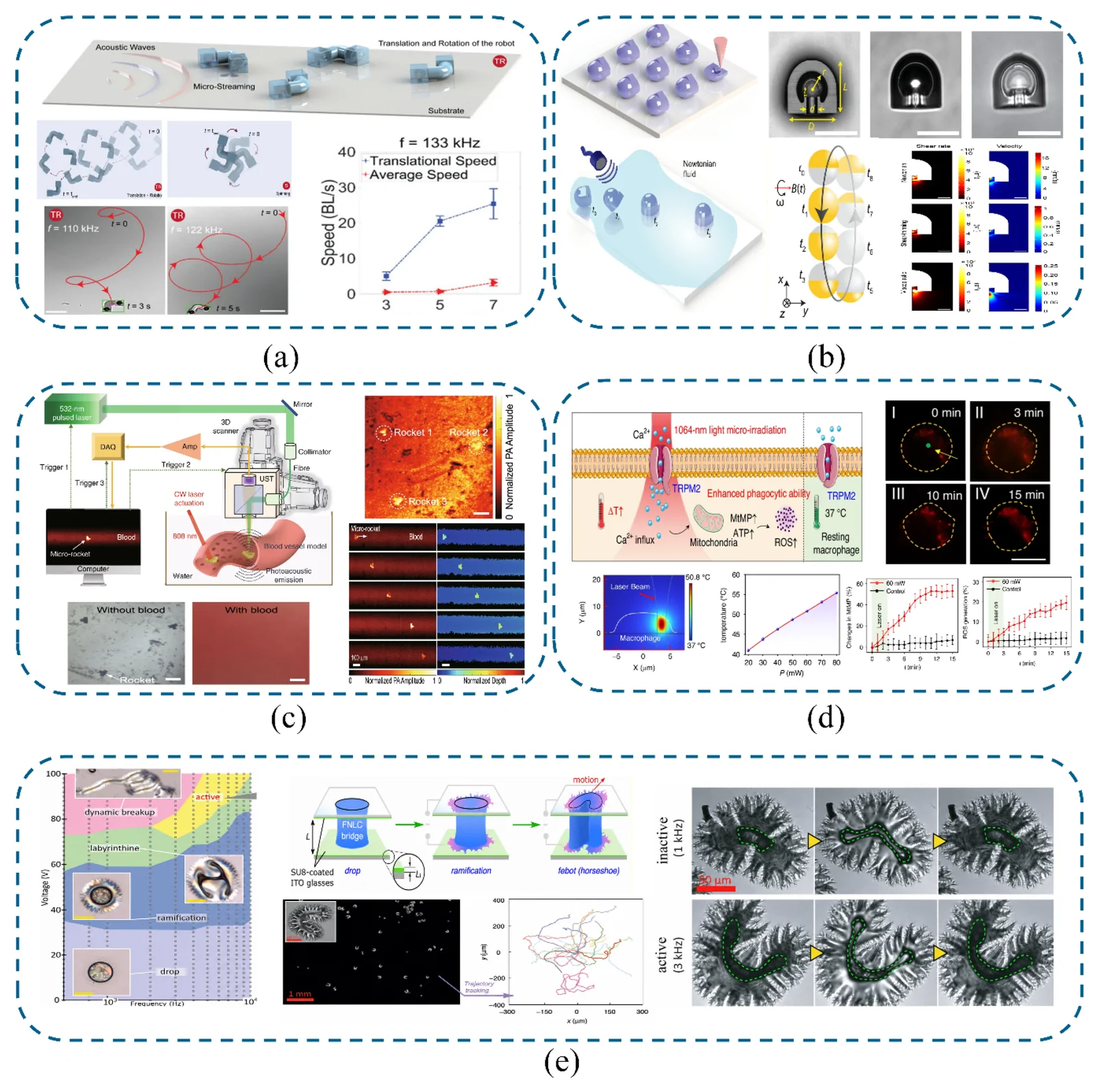
Physical actuation beyond magnetic fields. Acoustic, optical, thermal and electric inputs are organized by the work they perform in the task chain: multimode motion, propulsion in complex media, tracked actuation, local stimulation and soft-matter deformation. (**a**) Bubble-based acoustic microrobots use acoustic streaming to generate frequency- and amplitude-tuned rolling, spinning and translating motions [90]. (**b**) Trapped-bubble acoustic robots retain propulsion in Newtonian, non-Newtonian and mucus-like media, addressing complex fluid actuation without implying universal in vivo navigation [28]. (**c**) All-optic microrockets couple optical actuation with photoacoustic tracking in blood-related settings, linking motion generation to state visibility [92]. (**d**) NIR-activated phagocytic macrophage microrobots convert light input into local activation of a living-cell carrier, linking photothermal actuation with phagocytic task function [103]. (**e**) Electric fields reshape and move ferroelectric nematic droplets, converting soft-matter deformation into controllable locomotion states [93]. Image credits: panels (**a**), (**c**), (**d**) and (**e**) were adapted from Refs. [90], [92], [103] and [93], respectively, under the Creative Commons Attribution 4.0 International (CC BY 4.0) license. Panel (**b**) was adapted from Ref. [28] under the Creative Commons Attribution (CC BY) license.

Acoustic actuation becomes attractive in viscous and non-Newtonian biological fluids because ultrasound can deliver energy through tissue while the microrobot remains untethered. Bubble-based acoustic microrobots demonstrate the conversion step: frequency and amplitude tune acoustic streaming, with rolling, spinning and translation emerging as separate states [90] (Figure 7a). Trapped-bubble acoustic microrobots were designed to keep propelling at high-shear rates in complex biological fluids, directly addressing the performance loss that appears outside simple Newtonian liquids [28] (Figure 7b). Bioresorbable hydrogel robots add another constraint by linking acoustic propulsion with degradation and imaging-compatible operation in biofluids [40]. Dual ultrasound and photoacoustic tracking of magnetically driven micromotors showed that a tissue-permissive energy window can also locate moving micromotors in phantom and ex vivo settings before in vivo use [47]. Acoustic actuation is strongest when propulsion must remain visible during tissue-permissive energy delivery.

In microfluidic chambers, acoustic energy can do more than push a swimmer forward. It can deform a body or reorganize a collective state. Acoustic shape-morphing micromachines used an acoustic field for millisecond-scale reversible shape transformation, making deformation an actuation output rather than a passive design feature [101]. Magneto-acoustic field-induced microswarms combined magnetic and acoustic fields to form unstable collective structures; here acoustic input reshaped the swarm instead of simply driving individual particles [102]. A model-based learning controller for ultrasound-actuated microrobots brings the same input into closed feedback, particularly when the main uncertainty is steering through a changing fluid workspace [39]. These examples separate acoustic actuation from generic vibration. The useful output may be a swimmer’s propulsion, a body’s shape change, a swarm’s reconfiguration or a guided behavior.

Blood and living-cell settings favor light or heat when local precision matters more than whole-body penetration. An all-optically actuated microrocket used near-infrared light for motion in blood, while optical-resolution photoacoustic imaging located the robot [92] (Figure 7c). For single-cell thermal perturbation, a light-driven microrobot moved to the target and heated it while nanosensor feedback tracked the stimulus in space and time [104]. The light-powered phagocytic macrophage microrobot moves the example from synthetic single-cell heating to a living carrier. Its phagocytic activity can be activated by NIR illumination in vitro and in vivo [103] (Figure 7d). These systems are powerful local tools, but their window is set by optical access, heat management and the need to see or reach the target region.

Electric actuation is most useful in electrode-defined liquid chambers. There, local boundaries can be fixed and the field frequency or strength can be set directly. Ferroelectric nematic droplets activated by electric fields changed shape and translated through the chamber; polarization-driven morphology was the actuation mechanism [93] (Figure 7e). Light-stimulated micromotor swarms in an electric field combined illumination with electric polarization to tune spatial, temporal and mode control of silicon micromotor swarms [105]. This makes electric actuation a precise local control strategy for accessible or engineered workspaces. It should not be read as a universal deep-tissue substitute for magnetic or acoustic inputs.

Across soft tissue, blood, single-cell settings and electrode-defined chambers, physical-field actuation succeeds only when the field reaches the target and the body converts that input into the intended local effect. Acoustic inputs fit tissue-permissive energy delivery. Light and heat fit local stimulation or tracking. Electric fields work best where electrodes or accessible chambers can define the workspace. The design question is not which field is most advanced in isolation—it is which input still produces the required work in the chosen environment.

### 3.3. Chemical and Enzymatic Actuation in Reactive Working Environments

**Figure 8:**
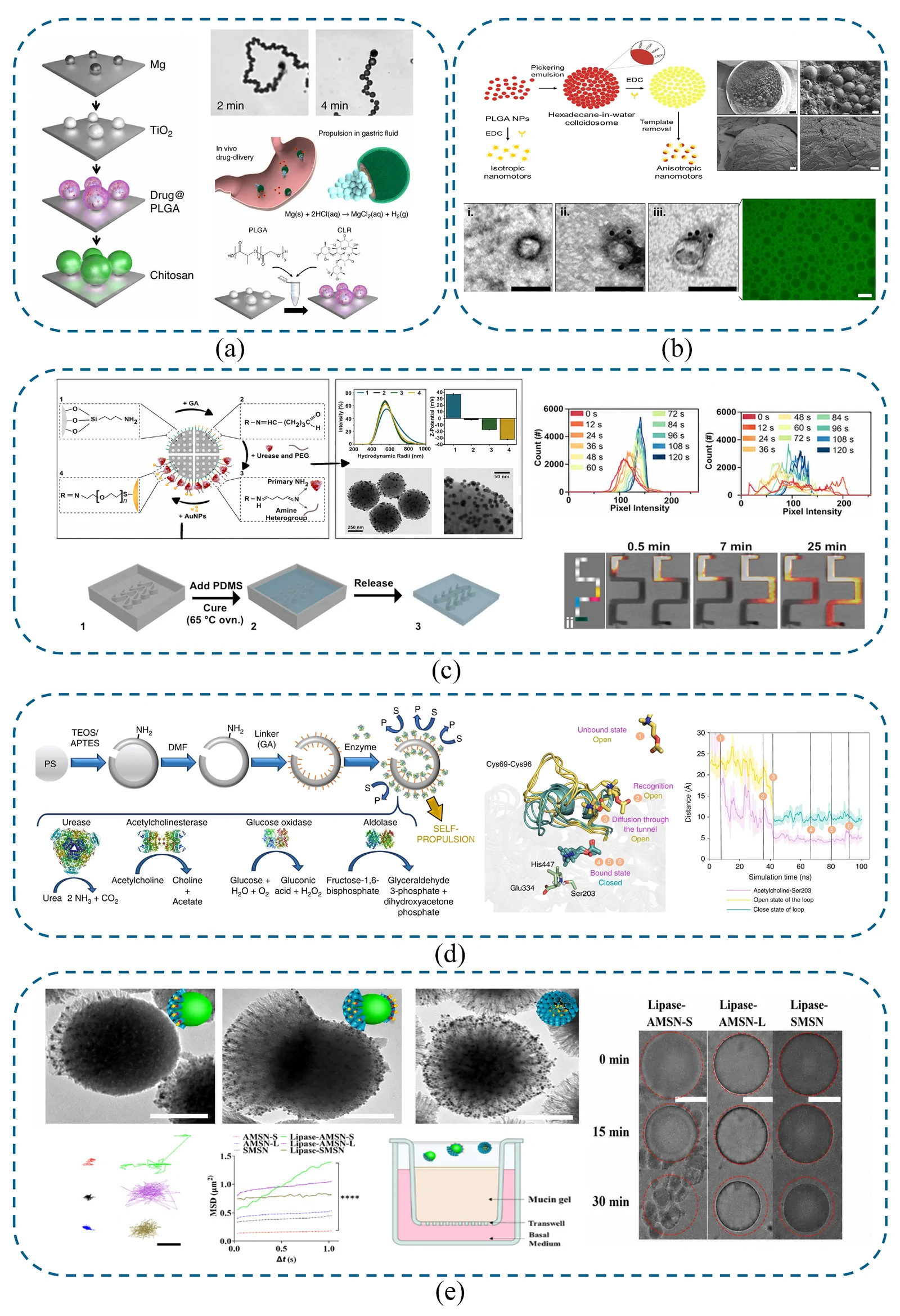
Chemical and enzymatic actuation in reactive working environments. Local fuel or substrate chemistry becomes task-relevant when it is converted into propulsion, release, collective accumulation, motion tuning or barrier crossing. (**a**) Gastric acid powers Mg-based micromotors while coupling propulsion to antibiotic release in the stomach [14]. (**b**) Engineered biodegradable urease-powered nanomotors use enzymatic urea conversion to improve gastrointestinal diffusion, distribution and localization [35]. (**c**) Enzyme-powered nanomotors form monitorable swarms or local accumulations in bladder-oriented settings, linking chemical actuation to readable distribution [106]. (**d**) Enzyme identity and intrinsic catalytic properties tune self-propulsion, showing that enzyme modules are not interchangeable actuation parts [94]. (**e**) Lipase-powered asymmetric nanomotors connect enzyme activity, lipid droplet degradation, motion and epithelial/transwell penetration, showing that mucus crossing depends on the matched enzyme–substrate pair [32]. Image credits: panel (**c**) was reproduced from Hortelao et al., Science Robotics, 10.1126/scirobotics.abd2823 (2021), AAAS, with permission (Ref. [106]). Panels (**a**), (**b**), (**d**) and (**e**) were adapted from Refs. [14], [35], [94] and [32], respectively, under the Creative Commons Attribution 4.0 International (CC BY 4.0) license.

In acid-rich gastric liquid, chemical actuation turns on reaction rate. The same fluid that threatens many materials can also supply protons for propulsion. Artificial micromotors in the mouse stomach established in vivo propulsion in gastric fluid, treating acid as an energy source rather than only a stressor [65]. Mg-based micromotors for stomach infection paired acid-driven motion with antibiotic release during *Helicobacter pylori* treatment [14] (Figure 8a). A gastric-fluid-gradient study adds an important qualification: pH and conductivity can switch the device between chemical propulsion, magnetic rolling and electrical operation, so local chemistry changes the available actuation mode [66]. Acid-powered systems should therefore report how long propulsion lasts, how gas generation is handled, whether payload release occurs, and what material state remains after reaction.

Urea-containing fluids provide a direct test of enzymatic actuation. Substrate conversion must produce motion that can accumulate or be imaged, not just a reaction on paper. Engineered biodegradable urease-powered nanomotors used urea conversion to support active diffusion and gastrointestinal distribution/localization, so substrate availability and post-administration localization became task-relevant outputs [35] (Figure 8b). In the bladder, urease-powered nanomotors displayed swarming behavior and could be monitored in vivo by positron emission tomography, linking enzymatic actuation with collective motion and tracking [106] (Figure 8c). Reaction-driven density changes in enzymatic nanomotor swarms also produced buoyancy, showing how many particles can be transported collectively [107]. Urease is therefore not merely a fuel module. Useful work depends on substrate level and product formation, then on whether the particles move collectively.

Enzymatic actuation in mucus-rich or diseased mucosal settings is valuable only when reaction-driven motion changes transport through the barrier or prepares local treatment. Urease-powered nanomotors enhanced mucosal and tumor penetration before sonodynamic therapy in bladder cancer, tying enzymatic motion to treatment preparation [30]. Catalase-powered nanobots used local peroxide decomposition to support intestinal mucus disruption and barrier crossing [31]. For this panel, lipase-powered asymmetric silica nanomotors give the cleaner mucus crossing example: lipase activity and lipid substrate degradation drive asymmetric motion tied directly to mucus and epithelial penetration [32] (Figure 8e). The enzyme choice matters because the target space must contain or tolerate the substrate, the product and the reaction rate.

Controlled enzyme panels make it hard to treat reaction-driven motion as one mechanism. In hollow silica micromotors, a systematic study showed that intrinsic enzymatic properties modulate self-propulsion, so enzymes are not interchangeable modules [94] (Figure 8d). A biomimetic sweeping microrobot for ulcerative colitis used Mg-based motion and surface functionality to remove pathogenic cell-free DNA and reactive oxygen species in colonic mucus [73]. Twin-bioengine self-adaptive micro/nanorobots combined enzyme actuation with macrophage relay for gastrointestinal inflammation therapy, placing reaction-driven motion in an immune and inflammatory setting [74]. Chemical and enzymatic actuation are strongest when a local reaction produces the needed action. Their weakness appears when substrate availability shifts, byproducts accumulate, or disease chemistry varies unpredictably.

### 3.4. Biological and Biohybrid Actuation Using Living Motility

**Figure 9:**
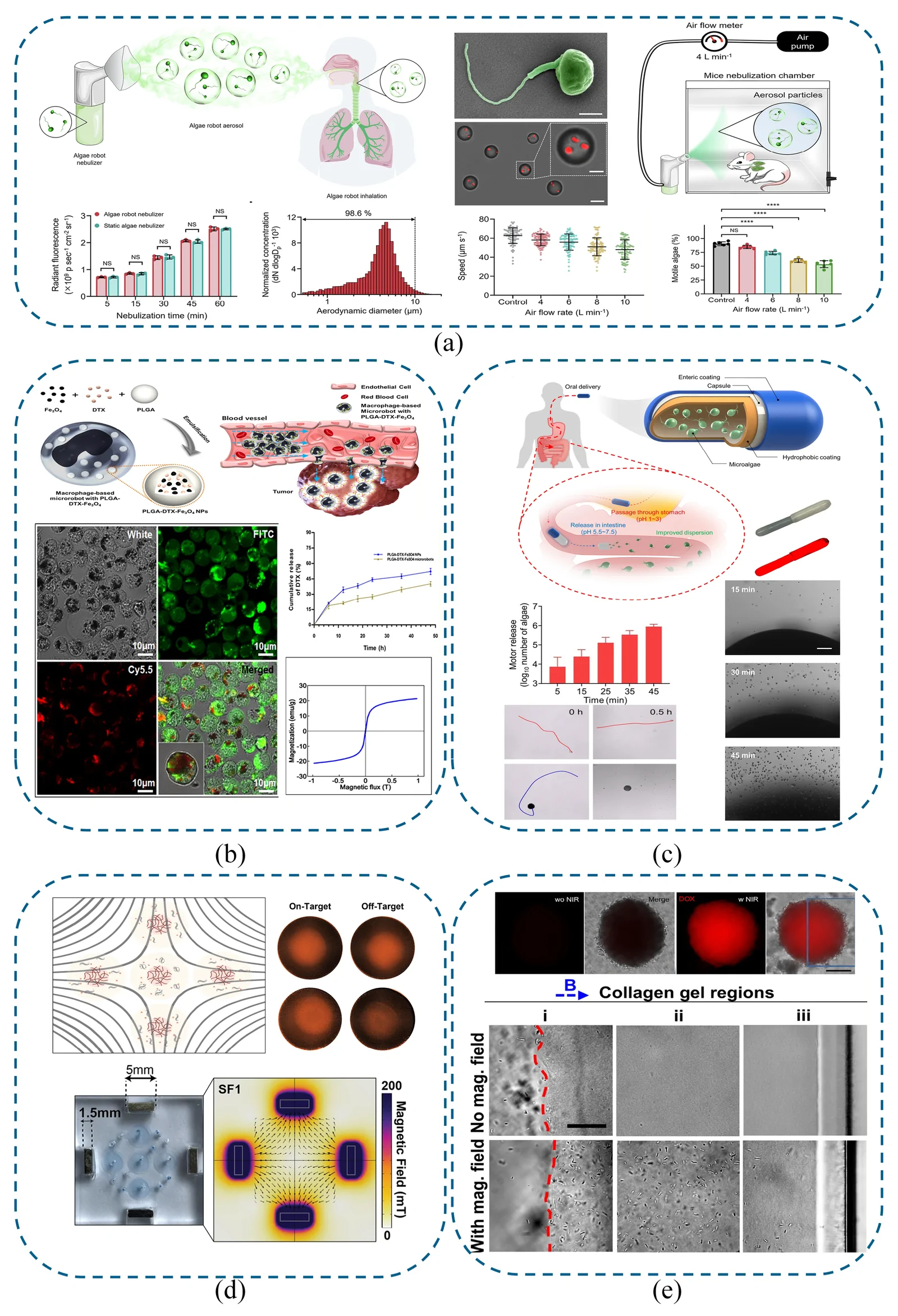
Living and biohybrid actuation using biological motility. Living motors become task tools only when their motility survives processing, loading, encapsulation, guidance or 3D matrix transport. (**a**) Inhalable algae biohybrid robots retain motility after nebulization and airflow exposure, supporting post-delivery pulmonary activity [17]. (**b**) Hybrid-actuating macrophage-based microrobots use macrophage uptake of drug/magnetic particles, magnetic targeting and tumor infiltration to turn an immune cell into an active therapeutic carrier [108]. (**c**) Enteric-coated degradable capsules protect algae motors during gastrointestinal entry and release motile cells under permissive intestinal conditions [109]. (**d**) Torque-focused magnetic delivery concentrates actuation in a selected spatial region; this panel is control-oriented and should not be described as bacterial biofilm targeting [111]. (**e**) Bacterial microrobots move through 3D biological matrices and release cargo under external stimulation, extending living actuation from planar transport to matrix delivery [95]. Image credits: panel (**c**) was reproduced from Zhang et al., Science Robotics, 10.1126/scirobotics.abo4160 (2022), AAAS, with permission (Ref. [109]). Panels (**a**), (**b**) and (**d**) were adapted from Refs. [17], [108] and [111], respectively, under the Creative Commons Attribution 4.0 International (CC BY 4.0) license. Panel (**e**) was adapted from Ref. [95] under the Creative Commons Attribution (CC BY) license.

Airway liquid asks a practical question of living motility: does the engineered carrier still move after processing and loading, then after deposition? Inhalable biohybrid microrobots used microalgae motility after nebulization to support non-invasive lung treatment, making post-deposition activity part of actuation [17] (Figure 9a). Nanoparticle-modified algae microrobots for acute bacterial pneumonia retained algae motion while carrying antibiotic-loaded nanoparticles [18]. Algae are not the only cell motors in this section. Hybrid-actuating macrophage microrobots internalized drug and magnetic components; after magnetic targeting, they infiltrated tumor tissue, making immune cell transport a cancer therapy delivery strategy [108] (Figure 9b). Algae-based biohybrid microrobots for lung metastasis used functionalized microalgae to actively deliver drug-loaded nanoparticles in the lung [19]. The central actuation question is whether the living motor remains motile after the engineering steps that make it therapeutic.

In intestinal liquid and inflamed tissue, biohybrid actuation has to survive protection and release before any relay step. Algae motors embedded in a degradable capsule exploited long-lasting swimming in intestinal fluid together with pH-sensitive release, improving local retention in the gastrointestinal tract [109] (Figure 9c). Twin-bioengine self-adaptive micro/nanorobots used enzyme-driven yeast capsules followed by macrophage relay to cross mucus and epithelium before immune cell transport steps in gastrointestinal inflammation [74]. These systems should be assessed after delivery. Encapsulation and release can alter motility and so can cell handoff.

Biofilms and biological matrices make bacterial actuation useful only when taxis and self-propulsion can be concentrated by magnetic guidance in a dense target. Magnetotactic bacteria-powered biohybrids targeted *E. coli* biofilms and released ciprofloxacin under acidic biofilm conditions [110]. The current Figure 9d is better read as a spatial selectivity example than as bacterial actuation. Torque focusing used magnetic field design to localize transport to a selected region, so it fits control-oriented delivery rather than biofilm-targeting living motility [111] (Figure 9d). Magnetically steerable bacterial microrobots integrated magnetic particles and drug-loaded nanoliposomes onto *E. coli* for movement in three-dimensional biological matrices and stimuli-responsive cargo release [95] (Figure 9e). Magneto-aerotactic bacteria carrying drug-containing nanoliposomes reached hypoxic tumor regions, turning a pathological chemical gradient into delivery guidance through bacterial taxis [112]. The limitation remains tied to the advantage: living bacterial engines bring biosafety and immune-response questions, plus growth control and manufacturing, into actuation design.

Reproductive-fluid-like and immune cell settings rely on living-cell functions that passive particles cannot reproduce. Sperm-driven micromotors were evaluated in oviduct fluid and viscoelastic formulations, showing that reproductive-fluid-like environments can reshape biohybrid motion [83]. Immune-cell-based microrobots used remote magnetic actuation while retaining antitumor activity; medical imaging made the living-cell contribution observable [26]. Engineered neutrophil biomotors exploit catalytic activity and native immune cell behaviors to carry nanodrugs and support thrombolysis [61]. Biohybrid actuation trades mechanical simplicity for environmental adaptability and biological function.

Across pulmonary, gastrointestinal, biofilm, reproductive-fluid-like and immune cell settings, biological actuation draws strength from carriers that already move, sense or interact in biological environments. Its weakness comes from the same source. Viability and heterogeneity affect the motor itself; immune recognition, payload loading and post-task fate affect translation. A living engine can make a microrobot more environment-aware, but it also makes the system harder to standardize.

## 4. Materials, Structures and Fabrication

Actuation is the input delivered to a microrobot. Motion is what remains after that input meets the body and its surroundings. Drag, walls, payload and tissue contact turn a command into displacement, rotation, anchoring or transport. At this scale, material choice cannot be separated from geometry or fabrication accuracy. A hydrolyzable PEGDA helical robot, for example, used disulfide-linked magnetic nanoparticles so that propulsion, DOX loading and post-delivery magnetic particle retrieval were designed into one body [113]. In GelMA microrobots made by flow-focusing droplet microfluidics, SPION loading and hNTSC attachment were combined with mass production in a degradable spherical carrier [114]. Hydrogel-enveloped porous microrobots for liver chemoembolization placed magnetic nanoparticles and therapeutic/imaging agents in a body that could be guided by X-ray, remain visible by postoperative MRI, and gradually degrade [115]. The question is therefore not which material or fabrication label is used. It is whether the resulting structure can keep motion and function reliable in its working environment and remain acceptable after the biomedical task.

Translation-facing constraints extend beyond making one body. Fabrication reviews identify sterilization procedures and application-dependent regulatory classification as unresolved issues for 3D-printed microrobots [7]. Mass production capability has been demonstrated for one biodegradable GelMA platform [114], but that platform-specific result does not establish uniform manufacturing, sterile packaging or approval readiness across microrobot systems.

### 4.1. Maintaining Motion: Preserving Actuation Output Through Body Design

**Figure 10:**
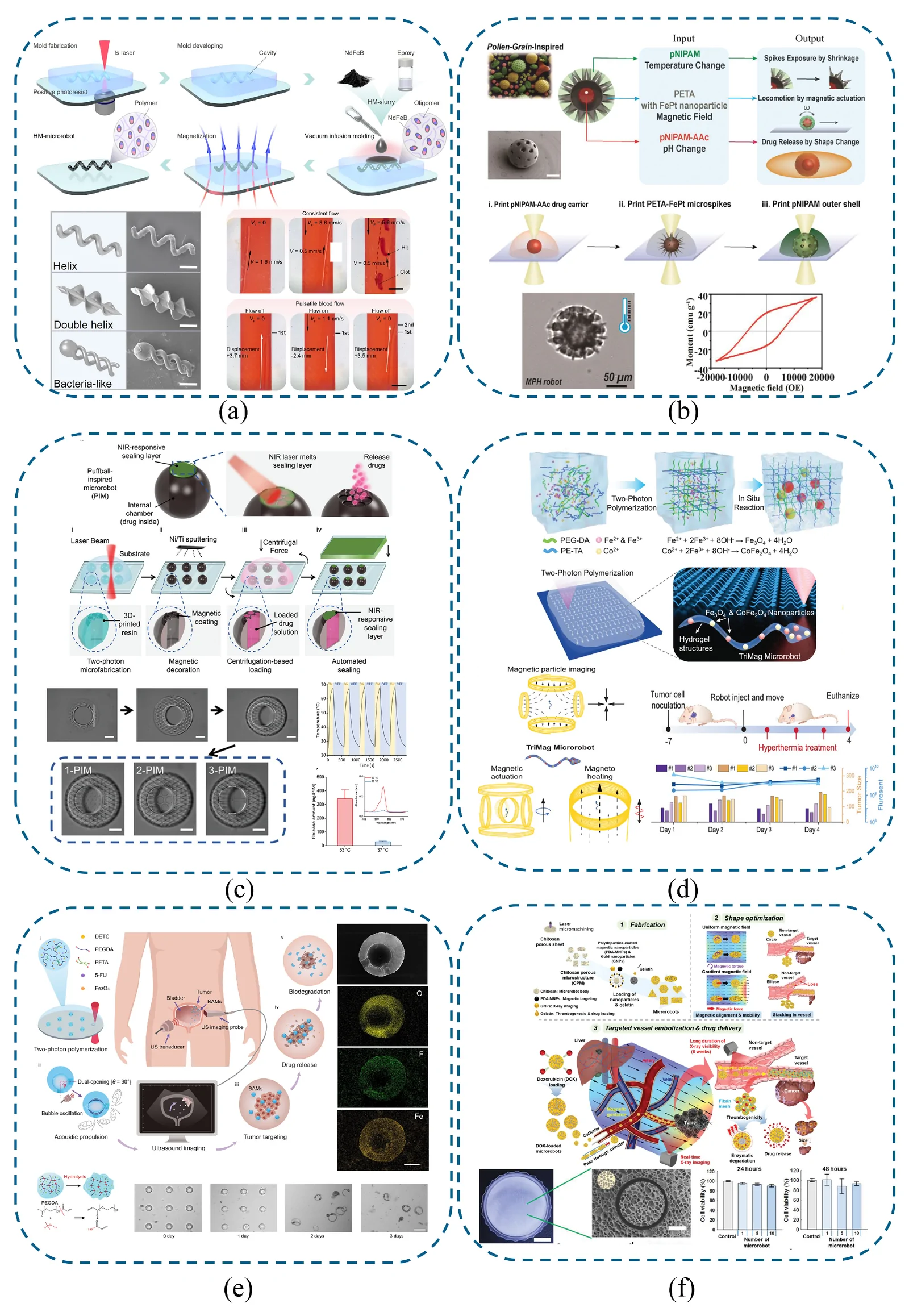
Body design for task execution. To maintain locomotion, enable specific biomedical functions and ensure biocompatibility, body-level solutions are developed through coordinated choices of materials, fabrication strategies and structural architectures. (**a**) Hard-magnetic TPP-VIM bodies preserve magnetic loading, morphology and upstream locomotion under blood-flow-like constraints [12]. (**b**) Pollen-inspired multihydrogel bodies decouple magnetic rolling, surface anchoring and pH-mediated cargo release after arrival [78]. (**c**) Puffball-inspired microrobots protect payloads during motion and open an NIR-responsive cap for triggered release [37]. (**d**) TriMag hydrogel microrobots integrate magnetic actuation, magnetic particle imaging and local hyperthermia into one body design [48]. (**e**) Bioresorbable acoustic hydrogel microrobots combine ultrasound-visible propulsion, drug release and post-task degradation [40]. (**f**) Biodegradable chitosan microrobots illustrate interventional body design for catheter delivery, vascular stacking, X-ray visibility, drug release and degradation [117]. Image credits: panel (**a**) was reproduced from Wu et al., Science Advances, 10.1126/sciadv.adw1272 (2025), AAAS, with permission (Ref. [12]); panel (**e**) was reproduced from Han et al., Science Robotics, 10.1126/scirobotics.adp3593 (2024), AAAS, with permission (Ref. [40]). Panel (**f**) was reproduced with permission from Go et al., A Soft Biodegradable Chitosan-Based Medical Microrobot with Optimized Structural Design and X-Ray Visibility for Targeted Vessel Chemoembolization, Advanced Functional Materials, doi: 10.1002/adfm.202305205; published by John Wiley & Sons, 2023 (Ref. [117]). © 2023 Wiley-VCH GmbH. Panels (**b**), (**c**) and (**d**) were adapted from Refs. [78], [37] and [48], respectively, under the Creative Commons Attribution 4.0 International (CC BY 4.0) license.

Magnetic bodies preserve motion when geometry and magnetic loading turn an applied field into a repeatable mechanical response. For Ni/Ti-coated SU-8 or IP-L helical micromachines, helix angle and diameter worked with coating geometry to govern magnetic alignment, wobbling and corkscrew propulsion. Under the same rotating field conditions, the reported 45-degree helix swam faster than several larger-angle designs [89]. The hard-magnetic microrobots printed using TPP-VIM reached the same design point through fabrication: a viscosity-adjusted NdFeB-loaded slurry yielded uniform helical bodies, while the D/d ratio and NdFeB mass fraction set the forward velocity and step-out frequency [12] (Figure 10a). In bioinspired soft microrobots for microvascular thrombolysis, non-swelling microgel bodies held aligned magnetic nanoparticle chains, so a rotating magnetic field produced controlled rolling–slipping motion rather than soft-body distortion [116]. These examples explain why magnetic material cannot be chosen apart from body geometry. The field supplies the input; the body supplies the mechanical path that makes useful motion possible.

Surfaces that touch tissue or flowing fluid must generate traction without trapping the robot permanently. A multilayer magnetic soft robot used patterned magnetic films together with adhesive interfaces, allowing one body to shift between mobile gaits and controlled attachment on wet tissue [29]. Claw-engaged swimming microrobots took another route: the clawed geometry supplied vessel retention, while the red-blood-cell membrane surface provided biological camouflage under flow [9]. In pollen grain-inspired hydrogel microrobots, motion and contact were assigned to different body regions. FePt nanoparticle-embedded PETA handled magnetic rolling. pNIPAM supplied temperature-responsive anchoring, and pNIPAM-AAc enabled pH-responsive cargo release [78] (Figure 10b). The design problem is therefore more specific than reducing friction. The surface and structure have to permit movement and contact before release, while keeping the robot controllable.

Fabrication enters motion control whenever a small change in size, particle distribution or magnetization changes the gait. In the GelMA droplet system, a flow-focusing microfluidic channel produced spherical biodegradable microrobots with measured size distribution and wrinkled surface morphology. The same work mapped SPION loading and then demonstrated magnetic actuation of hNTSC-loaded microrobots in a confined channel [114]. The TPP-VIM hard-magnetic design showed this dependence from another angle. Low slurry viscosity led to separated structures, whereas optimized prepolymerization gave a uniform NdFeB distribution and allowed multiple helical morphologies [12]. Modular magnetic microrobots for endoluminal navigation carried the manufacturing problem into a larger medical tool format, using detachable magnetic modules to maintain navigation and radial force during stent delivery in complex ductal anatomy [91]. For motion-preserving body design, fabrication should be judged by whether it reproduces the features required by the actuation mechanism, not by resolution alone.

Manufacturing repeatability is part of the field-to-motion link. The TPP-VIM hard-magnetic study connects NdFeB loading and helical geometry with propulsion [12]. The GelMA flow-focusing study reports size distribution together with a mass production capability [114]. A small but important reporting gap remains: common batch-to-batch statistics for particle distribution and helical dimensional tolerances are not provided, so these values should be reported in future studies rather than inferred here.

Geometry and magnetic loading handle the field-to-motion link; softness and surface contact handle tissue or wall interaction; fabrication reproducibility handles whether that solution can be made again. Maintaining motion is therefore a body design constraint, not a stand-alone material choice. The useful biomedical body is the one whose added complexity still leaves a predictable link between input, structure and movement.

### 4.2. Adding Function: Payload, Sensing and Therapeutic Activity Without Losing Control

Payload function is credible only if loading does not break navigation or blur the release event. Puffball-inspired microrobotic systems used an internal chamber to hold a large cargo volume and a NIR-responsive sealing layer to protect it until release; a magnetic coating retained targeted locomotion [37] (Figure 10c). In the fish-inspired multidrug delivery microrobot, the 3D-printed hydrogel body kept ASA and DOX in separate regions. This arrangement allowed transport to remain linked to staged release and synergistic cancer-cell treatment, instead of turning the robot into a single drug depot [118]. Magnetic hydrogel soft capsule microrobots formed a hydrogel membrane from CaCl_2_-containing suspension droplets and magnetic powders in sodium alginate. That membrane enclosed microrobot swarms and cargo, and a stronger gradient magnetic field physically disintegrated the capsule for release [119]. Here, body design protects the payload during travel and defines how release occurs. It is not just empty storage space.

Functional parts have to be placed so they do not overpower the actuation body. Imaging, therapy and sensing are useful only when their hardware has a defined job inside the moving structure. TriMag microrobots generated magnetic nanoparticles inside a 3D-printed PEGDA/PETA hydrogel, so the helical body could actuate magnetically while also supporting magnetic particle imaging and magnetothermal heating [48] (Figure 10d). For chemoembolization, a hydrogel-wrapped porous microrobot carried magnetic, therapeutic and contrast components in one structure. This arrangement let the operator guide placement by X-ray feedback and assess distribution by MRI afterward [115]. Swarming magnetic photonic crystal microrobots used pH-responsive hydrogel microspheres with encapsulated magnetic components, making local pH visible and coupling that signal to drug delivery [120]. A drilling-sensing microrobot for tracheal microlesions placed a gold nanospike tip at the working end for tissue sampling and SERS biosensing; magnetic navigation and retrieval remained under tracheoscopy or fluoroscopy [121]. A functional body is useful when a contrast agent, heater, sensor or sampling tip occupies a defined position and performs a defined task inside the moving structure.

Other designs add function by making the carrier modular or by using a biologically active body. Modularized bile-duct microrobots split magnetic actuation from the cell-scaffold module. Their pH-responsive deformability and lock-detachable design reduced the trade-off between magnetic responsiveness and cell loading-release capability [122]. Ionic shape-morphing alginate end-effectors encoded hydrogel crosslinks through electrode configurations, pairing magnetic manipulation with environment-responsive targeting, release and sampling [123]. Magnetically steerable bacterial microrobots used *E. coli* bodies carrying magnetic nanoparticles and DOX/ICG-loaded nanoliposomes. In that carrier, biological motility supported magnetic steering and NIR-triggered drug release [95]. Biomimetic magnetobacterial microrobots for pneumonia therapy likewise treated bacterial body function as part of the therapeutic design rather than as add-on cargo transport [124]. Modular and biohybrid bodies work by separating functions within the structure. The price is a larger design problem that includes viability, module detachment, loading consistency and biological containment.

### 4.3. Biomedical Acceptability: Compatibility, Degradation, Retrieval and Residue

Biomedical acceptability turns on the body material, the released species and the residue left after the task, not on a single biocompatible label. In the Mag-C system, a magnetic chitosan microscaffold combined programmable morphology with surface-loaded magnetic nanoparticles, tying chitosan biodegradability to body shape and magnetic responsiveness [36]. The soft biodegradable chitosan-based chemoembolization microrobot used a porous chitosan sheet with attached PDA-MNPs and gold nanoparticles. Gelatin loading and optimized shapes supported magnetization and vessel stacking [117] (Figure 10f). Imaging-guided bioresorbable acoustic hydrogel microrobots linked propulsion to post-task fate: ultrasound supported real-time tracking, while hydrolysis drove degradation of the hydrogel body [40] (Figure 10e). In these cases, degradation is body-specific, not a generic material label.

Compatibility is stronger when degradation products and tissue response are tested rather than inferred from the material name. The magnet-driven degradable stem-cell-delivery microrobot used a burr-like porous PEGDA/PETA hydrogel body. Its degradation products were tested by MTT assay against therapeutic cells, normal hepatocellular cells and hepatocellular carcinoma cells [125]. The GelMA magnetic microrobot tested hNTSC viability after attachment and magnetic actuation, then connected enzymatic GelMA degradation with cell release and later neural differentiation [114]. The chitosan chemoembolization microrobot measured HUVEC viability and lysozyme-mediated degradation while using gelatin loading to promote thrombogenic vessel blocking [117]. An acceptable body is therefore evaluated through material response and degradation products in the context of biological outcome.

Retrieval offers a different route for managing residue when degradation is incomplete or too slow. The PEGDA helical microrobot with disulfide-linked magnetic nanoparticles used DTT- or NIR-triggered magnetic particle separation after DOX delivery, so the magnetic fraction could be retrieved instead of remaining with the drug carrier [113]. In an ocular hydrogel-sheet design, the therapeutic layer and magnetic component were arranged so delivery could be followed by nanoparticle recovery as a separate post-task step [126]. A later helical microrobot combined magnetic particle retrieval with sequential dual-drug release, separating FUS-triggered MNP removal from the NIR- and degradation-mediated release steps [127]. The magnetically guided self-rolled microrobot made retrieval a system function. X-ray imaging, EMA navigation and local drug release were tied to recovery through a coiled catheter [99]. Retrieval evidence should therefore be reported as retrieval, not biodegradation. It answers a different post-task safety question.

These examples also expose distinct failure boundaries. Loss of wall contact under flow is a retention failure [9], whereas incomplete degradation or residual magnetic material is a post-task residue problem. The cited systems demonstrate hydrolysis-driven degradation of a bioresorbable hydrogel body, magnetic component separation and X-ray-guided catheter recovery as different task-specific exit routes [40,99,113,126,127]. These demonstrations do not establish universal rates of embolization, inflammatory response, degradation debris occlusion or macrophage clearance. A safety assessment should therefore report the failure trigger, whether the robot remains observable, and the recovery, degradation or containment route tested for that task.

Biological coatings and living bodies can improve task compatibility, but they shift part of the design burden to biological control. Zwitterionic 3D-printed stealth microrobots used antifouling surface chemistry to reduce protein and immune contact, treating surface chemistry as a body design variable rather than a passive coating label [45]. In claw-engaged microrobots, the red-blood-cell membrane created a biolubricated and camouflaged vascular surface, while the claw geometry supplied mechanical retention [9]. Algae-based biohybrid microrobots retained living motility while carrying therapeutic nanoparticles for lung applications [18]. Bacterial microrobots add taxis and drug delivery. The living *E. coli* body also brings viability and immune interaction into the body design problem, together with growth control and post-treatment containment [95]. Biological compatibility is therefore not one material property. It is a body-specific question about survival, interaction and removal.

Body design links actuation to biomedical use by determining what the robot can do, how it performs and what is left afterward. A material, structure or fabrication method matters only when its contribution can be traced to motion preservation, functional integration or post-task acceptability. This body-centered view leads to the next problem: the same objects must be localized and tracked while those functions are being performed, then controlled from the measured state.

## 5. Detection and Control: From Task State to Action

A biomedical microrobot does not become useful simply by reaching a site, moving there and carrying a functional body. The task must also be visible to the experimenter. Position and motion have to be separated from the biological background, as do post-contact events such as sampling, release and degradation. Current readouts fall into several families. MRI, X-ray/CT or fluoroscopy and ultrasound provide deep anatomical or interventional views; photoacoustic/optoacoustic imaging and magnetic particle imaging add contrast from robot components; optical coherence tomography, fluorescence imaging, nuclear imaging and image-processing-based tracking cover more local or signal-specific cases. These methods differ in penetration, spatial and temporal resolution and contrast source [4]. Ultrasound-focused work makes the same point in practical terms: a single robot and a swarm pose different localization and signal-detection problems, so the method must match the object being followed [128]. Control uses the measured state to adjust a magnetic, acoustic, optical or electric input. In most microrobot systems, an external field generator and controller make that decision rather than an on-board processor [5]. Table 2 separates reported depth, detection or spatial metrics, acquisition performance and drive compatibility while preserving whether each result was obtained in a phantom, ex vivo or in vivo.

### 5.1. Detection: Measuring Task-Relevant States

**Figure 11:**
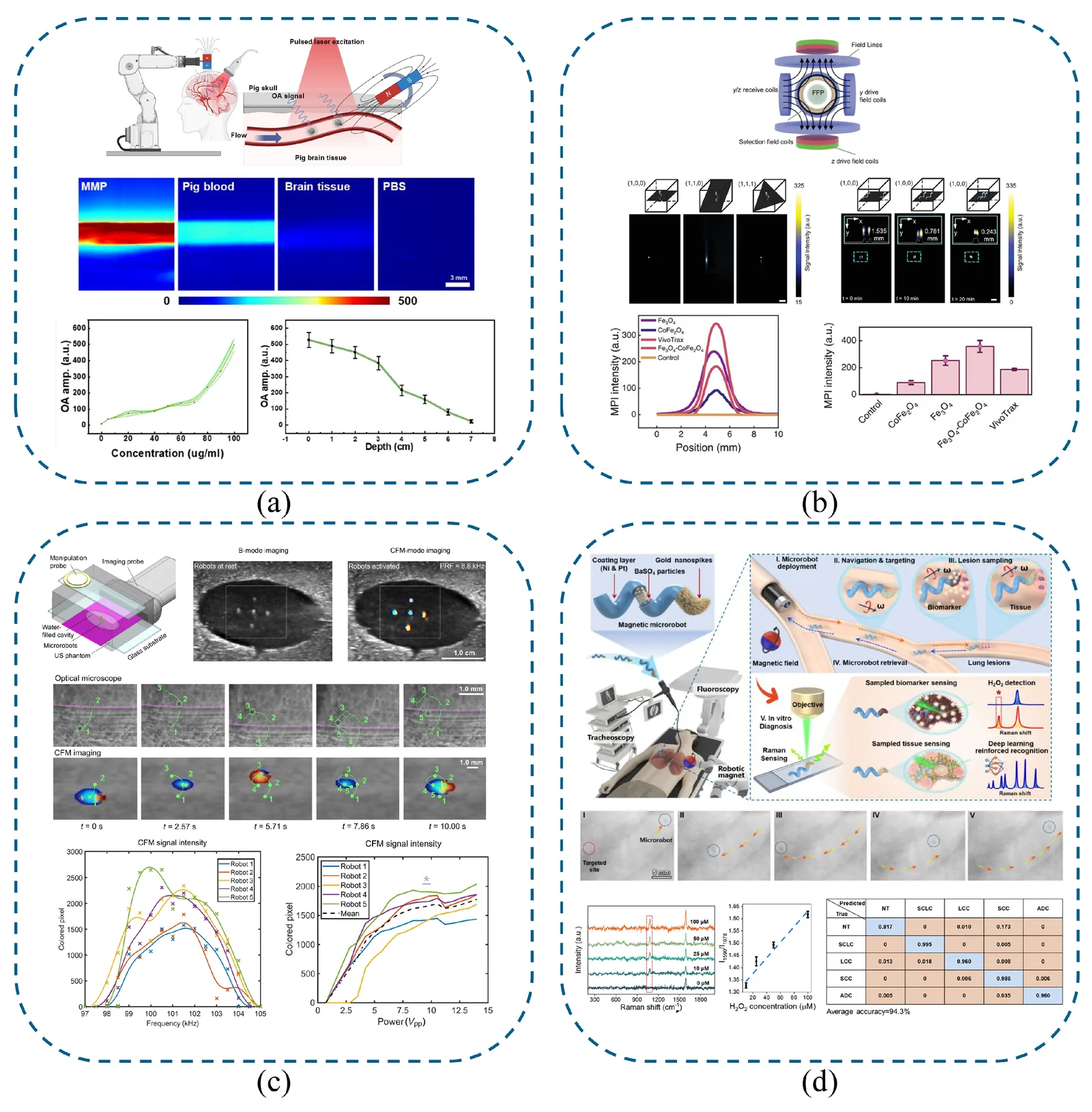
Detection as state readout. Detection closes a task gap only when it converts microrobot location, motion, body signal or sampled material into a measurable state that can support judgment or control. (**a**) MSOT/optoacoustic imaging reads depth-dependent position and flow-channel motion through blood and tissue barriers [38]. (**b**) Magnetic particle imaging localizes magnetic microrobot bodies and distinguishes adjacent robots, making the body itself an imageable state [48]. (**c**) Color-flow ultrasound maps pseudo-Doppler signals into real-time microrobot trajectories, turning motion into a readable path [129]. (**d**) Biopsy/SERS microrobots extend readout beyond coordinates by sampling lesions and converting chemical or tissue information into diagnostic Raman signals [121]. Image credits: panel (**d**) was reproduced with permission from Wang et al., Magnetic Microrobot with Drilling-Sensing Dual Functionality for Targeted Biopsy of Deep-Seated Tracheal Microlesions, Advanced Materials, doi: 10.1002/adma.202514664; published by John Wiley & Sons, 2026 (Ref. [121]). © 2025 Wiley-VCH GmbH. Panels (**a**) and (**b**) were adapted from Refs. [38] and [48], respectively, under the Creative Commons Attribution 4.0 International (CC BY 4.0) license. Panel (**c**) was adapted from Ref. [129] under the Creative Commons Attribution (CC BY) license.

Deep or opaque settings first need contrast that separates the robot from tissue. Photoacoustic and optoacoustic imaging work when the robot, or one of its components, absorbs light and emits an acoustic signal. In an optoacoustic-guided FePt/ZIF-8 magnetic microrobot system, multispectral optoacoustic tracking followed reconfigurable swarms during vascular drug delivery experiments [38] (Figure 11a). Dual ultrasound/photoacoustic tracking split the same experiment into two readouts. Ultrasound provided tissue anatomy, while photoacoustic contrast marked the magnetically driven micromotors [47]. Magnetic particle imaging addresses a different limit. It detects magnetic tracers without optical transparency, as shown by TriMag, where a 3D-printed magnetic body could be localized while also supporting magnetic actuation and magnetothermal heating [48] (Figure 11b). X-ray and fluoroscopy become more persuasive when the task resembles an intervention. FePt surface microrollers were developed for medical-imaging-guided endovascular delivery. Self-rolled microrobots combined magnetic guidance with real-time X-ray imaging and catheter-assisted retrieval [56,99]. Liquid-metal microrobots added radiopacity and deformability for fluoroscopy-guided transvascular navigation. Covalent-organic-framework microswimmers paired light-driven therapy with photoacoustic and optical coherence tomography observation [131,132].

Motion tracking is a time-series problem, not a request for one visible frame [128]. Ultrasound can provide that record in more than one way. Acoustically stimulated bubble microrobots produced pseudo-Doppler color-flow signals for activation and localization in phantoms and an ex vivo bladder model [129] (Figure 11c). A miniature helical robot used ultrasound Doppler tracking during navigation in dynamic blood flow for thrombolysis [133]. Laser speckle contrast imaging fits a swarm-level state better than a single-body coordinate because it follows position and area together with morphology during targeted delivery [49]. In crowded or low-contrast videos, detection depends on image analysis as much as imaging hardware. MEMTrack used motion-enhanced inputs and a multi-level detector, then applied modified SORT tracking to extract bacterial micromotor trajectories in collagen [134]. Ultrasound detection benchmarks then ask whether AI classifiers can still find the robot signal under imaging noise. At the microscope scale, fluorescent light-field microscopy reconstructs high-speed three-dimensional trajectories [135,136]. Near-infrared II fluorescence navigation brings high-contrast localization into live animal experiments. Its strength is real-time image-guided localization, not evidence that the whole therapy is autonomous [130].

Diagnostic and sensing tasks shift the object of detection. The signal is no longer only the robot’s position; it is the local condition, analyte or captured target. Swarming magnetic photonic crystal microrobots used structural color from pH-responsive hydrogel microspheres to read local acidity before regulating drug delivery. Magnetic microrobots with fluorescent internal memory stored intragastric acidity as a recoverable chemical history [69,120]. Other systems turn motion into assay information. A real-time micromotor probe linked micromotor behavior to neutrophil activation, and digital micromotor tracking-enabled immunoassays treated many trajectories as quantitative assay signals [137,138]. Mechanical readout offers another route. Vibrating microrobot-induced elastography is best described as stiffness sensing for bowel cancer detection, not as a finished screening technology [139]. Contact-based diagnostic systems are more local and more invasive. A drilling-sensing microrobot used a gold nanospike tip for tissue drilling followed by SERS readout [121] (Figure 11d). A micromotor-functionalized DNA tile system captured circulating tumor cells. A magnetic-electric hybrid micromotor coupled propulsion with label-free cargo transport and sensing in conductive liquid [140,141].

Therapeutic carriers raise one more detection question: did release, deformation or degradation occur after actuation? Soft magnetic microrobots can embed remote readout and communication in deformable magnetic materials along with locomotion [142]. Four-dimensional printed microrobots used diffractive optical sensing and pH-adaptive morphing, so body deformation became part of the readout [143]. Single-cell microgel microrobots combined magnetic transport with light-responsive cell release; dual-mode T1/T2 magnetic-resonance readout then verified delivery [144]. Soft capsule rolling microrobots combined multimodal imaging with drug delivery and triggered degradation, so post-action fate could be followed after delivery [145]. The readout-to-control hierarchy is practical rather than abstract. Imaging locates the robot and tracking measures motion. Diagnostic readouts measure local biology. Fate readouts ask what happened after the therapeutic action. Control frameworks determine whether any of those readouts can guide the next input [4,5].

Existing studies demonstrate modality-specific workflows rather than a single three-modality system. A multifunctional microrobot was guided by X-ray and evaluated by postoperative MRI [115], while dual ultrasound and photoacoustic tracking localized magnetically driven micromotors [47]. A self-rolled microrobot combined real-time X-ray imaging, targeted delivery and catheter-assisted retrieval [99]. These examples support a proposed task state architecture: modality-specific acquisition feeds registration to a common spatial and temporal frame; an uncertainty-aware state estimator then passes task-relevant state to a safety and command-arbitration layer, which selects an actuation-specific command and a fallback action when a modality loses visibility [5]. Because the cited studies do not demonstrate simultaneous MRI–ultrasound–X-ray fusion, a practical clinical workflow should be treated as sequential or selectively concurrent rather than as established continuous three-modality operation.

### 5.2. Control: Using Measured State to Change Actuation

The control loop is iterative: a measured robot state selects the next command, the field is applied, and the state is measured again [5]. In most cases the controller sits outside the robot, since a microrobot normally cannot carry enough onboard processing, power and sensing hardware [5]. Magnetic systems show how this works. Six-degree-of-freedom magnetic control used pose and orientation feedback to command a single magnetic microrobot. A disturbance-aware electromagnetic controller incorporated force disturbance and localization uncertainty when computing magnetic inputs [50,88]. Acoustic control can be state-guided without being fully autonomous. In human-in-the-loop acoustic manipulation, the operator observed the microrobot and adjusted the acoustic actuation [146]. Automatic docking of magnetic helical microrobots gives a more algorithmic example: path planning and path-following control were separated from alignment and insertion [147].

Latency is a property of the complete loop, not of the imaging or learning module alone. The chain includes image acquisition, reconstruction or transfer, state detection, model inference, planning, command update and actuator response. The reported evidence is heterogeneous: one in vivo photoacoustic/ultrasound study acquired each imaging mode at 20 frames s^−1^ and used image feedback for PID control [125], whereas the model-based reinforcement-learning study used camera acquisition at 6–18 fps and described millisecond-scale PZT switching [39]. These are component-level observations, not end-to-end latency measurements. Other studies report acquisition settings such as 5–20 fps for HFUS/photoacoustic imaging, 100 Hz for volumetric MSOT and approximately 50 fps for NIR-IIb imaging [38,47,130], but do not establish a common closed-loop update rate. Future control studies should report the full-loop latency, jitter, update frequency and actuator response under the relevant fluid and imaging conditions, while keeping acquisition rate distinct from control performance.

**Figure 12:**
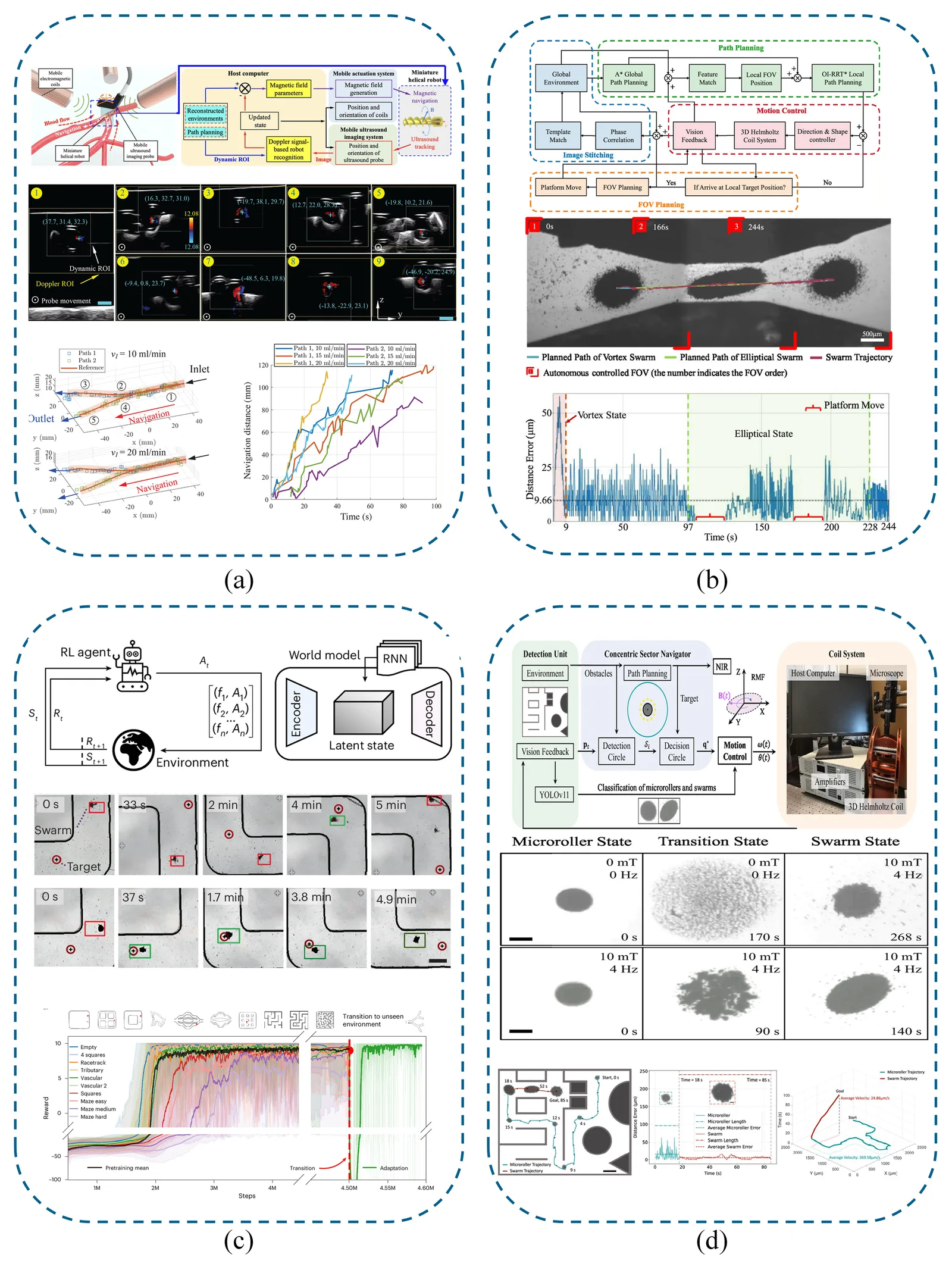
Control as state-to-action conversion. Control is treated as the step in which measured robot, image or swarm states determine the next field, platform or algorithmic action. (**a**) Ultrasound Doppler tracking and dynamic ROI recognition update magnetic navigation commands for a helical robot under flow [133]. (**b**) Autonomous field-of-view planning couples image stitching, path planning, platform motion and swarm control to keep a microswarm observable during long-range navigation [51]. (**c**) Model-based reinforcement learning maps image-derived states into ultrasound actuation policies through a learned world model [39]. (**d**) Vision-based dynamic obstacle avoidance combines YOLOv11 state recognition, concentric sector navigation and dynamic motion control decisions to convert visual state into obstacle avoidance action [148]. The asterisks in the figure belong to algorithmic and control notation rather than superscript markers: A* is the A-star search algorithm; OI-RRT* is an RRT* (optimal Rapidly-exploring Random Tree) local path planner; and q* denotes the selected optimal control command. Image credits: panel (**a**) was adapted with permission from Ref. [133]. Copyright 2022 American Chemical Society. Panels (**b**), (**c**) and (**d**) were adapted from Refs. [51], [39] and [148], respectively, under the Creative Commons Attribution 4.0 International (CC BY 4.0) license.

Closed-loop control requires the measured state to shape the next command, not simply appear on a display [5]. This distinction matters when the scene moves or the robot can leave the observable region. In dynamic blood flow, ultrasound Doppler tracking was coupled to autonomous navigation of a miniature helical robot for thrombolysis. Steering commands were updated from a moving state rather than a fixed planned path [133] (Figure 12a). Autonomous field-of-view planning addressed the opposite failure, loss of observation. The system stitched global images, planned with A* and OI-RRT*, chose the next observation window and monitored channel width with swarm position through visual feedback [51] (Figure 12b). Selective actuation adds spatial and temporal control. Torque focusing used rotating and selection magnetic fields to concentrate torque density in the intended region. Sequential magnetic-acoustic CAR T cell microrobots used magnetic navigation first and acoustic activation later [25,111]. Active microgel particle swarms for intrabronchial delivery make the same control need concrete in a branching airway-like geometry: collective motion has to be guided before local delivery can be assessed [77].

Swarm control and learning-based control are useful only after the controlled state has been stated [39,54]. For vascular network exploration, the reconstructed network served as information for later motion. For confined transport, artificial microtubules formed by swarm unraveling used collective morphology for directed cargo transport [86,87]. Other systems make the controlled variable broader than a single coordinate. In geometry-constrained navigation through cerebral bifurcations and in mass-produced programmable magnetic assemblies, the target state may be a path choice, a distribution or a swarm morphology [59,149]. Algorithmic control then becomes a state-to-action mapping problem [39,53]. Vision-based dynamic obstacle avoidance gives a concrete panel example: YOLOv11 recognition identifies microroller or swarm states, and a concentric sector navigator converts detected target and obstacle geometry into local motion decisions [148] (Figure 12d). Phase-diagram-guided process control used swarm shape and orientation for reconfiguration and trajectory tracking [54]. AI-assisted multimode swarm analysis classified collective behavior modes before field decisions were made [55]. Real-time distribution planning converted measured swarm distribution into navigation decisions [53]. Model-based reinforcement learning for ultrasound-driven microrobots used image-derived state, a learned dynamics model and frequency/amplitude actions [39] (Figure 12c). Reinforcement learning for three-dimensional magnetic positional control mapped observed position error to magnetic actions. Automatic control of magnetic diatom biohybrid microrobots linked image detection of microrobots, targets and obstacles to path planning and trajectory tracking [150,151]. Smart magnetic microrobots trained by deep reinforcement learning provide laboratory evidence for state-action learning. They should not be read as direct evidence of autonomous therapy [52].

## 6. Discussion and Outlook

A task-oriented view should judge biomedical microrobots by the fit between a biomedical problem and the small-scale actions used to address it. A useful system must first reach, or be placed in, the relevant biological setting. It then has to keep moving and contacting the target under local constraints. The body must preserve function, the task state must be measurable, and that state must inform the next action. Across the examples reviewed above, access and working environment lead into actuation, body design, detection and control as one task chain. Continuity is the important test. If the links hold together, a local microrobot action can become a biomedical operation. If one link fails, even an impressive device remains a partial demonstration rather than a task-ready biomedical system.

Clinical translation also requires a predefined response to task failure. In the studies reviewed here, hydrolysis-driven degradation of a bioresorbable hydrogel body, magnetic particle retrieval and X-ray-guided catheter recovery represent distinct routes for reducing or managing residual material [40,99,113,126,127]. They should be interpreted as platform-specific demonstrations rather than a universal clearance strategy. Before clinical use, each system should define how loss of control, off-target deposition, incomplete degradation and residual biological response will be detected and managed.

The chain starts with access conditions, not with an abstract robot type. Vascular tasks expose a device to flow and blood cells before vessel-wall contact, clot architecture, imaging depth or retrieval can be judged. Gastrointestinal and mucosal tasks begin with acid and transit, then become problems of mucus, diseased tissue and local retention. Inhaled or surface-facing tasks bring deposition and wet contact into view, followed by coverage, debris and removal. These conditions define useful work. Magnetic torque and acoustic streaming, light-driven deformation and electric field polarization, chemical propulsion and living motility matter only when they produce a task-relevant action in that setting. Body design carries the action through biological contact. Geometry helps preserve motion; materials and coatings manage adhesion and compatibility; cargo structures maintain loading and release; degradable or retrievable components define what remains after the task. Detection and control close the loop by turning measured state into the next field, stimulus or decision. That measured state may be position, speed, orientation, morphology, release, degradation, analyte signal or sampling output. The strongest studies do more than add functions. They show access, useful work and body contact feeding into state readout and control inside a chosen biomedical setting.

Future studies should make the task standard more explicit. Model choice is the first place to improve. Blood-facing systems should retain flow and cellularity, then test wall interaction and retrieval. Oral and mucosal systems should retain the chemical and barrier features that determine transport and residence. Biofilm and surface tasks should retain contact geometry first, then coverage, debris and post-cleaning residue. Comparison is the second place. A drug delivery microrobot should be judged against the relevant free drug or passive carrier, and also against catheter or local administration when those are the real alternatives. A cleaning robot should be compared with antimicrobial, washing or mechanical-removal methods. A sensing robot should be compared with the relevant imaging or diagnostic readout. The comparator should follow the task step being claimed, not the name of the actuation mode, material family or robot category. Post-task fate is a third evaluation dimension and should be considered as part of overall task performance rather than as a separate issue. Degradation and bioresorption answer one residue question; retrieval, residue toxicity, immune interaction and biological persistence answer others in the same environment in which the task was performed. A system that reaches a site and releases a payload remains incomplete if it cannot be tracked, cleared, degraded or justified against existing methods. Conversely, a simpler microrobot can still matter when it solves one difficult local step better than current tools and its fate after that step is understood.

The field does not need every future microrobot to look more complex. It needs better task-matched evidence. The most convincing studies will show access reaching the intended environment, input energy becoming useful action, body design preserving biological function and state readout changing a control decision. Biomedical microrobots are unlikely to replace broad medical tools. Their main promise is narrower: they may help complete local tasks in settings where conventional approaches struggle to combine arrival and retention with action, observation and adjustment within a single small-scale task.

## Figures and Tables

**Table 1:** Source-verified quantitative comparison of representative actuation studies.

Actuation Mode and Representative Study	Penetration, Input and Control Response	Force/Torque or Task Output	Physiological Compatibility and Damage Evidence
Magnetic; modular endoluminal microrobot [91]	Tissue penetration depth and formal spatial/temporal control resolution: NR. Rotating fields of 5 mT at 20 Hz or 10 mT at 30 Hz were tested in a 1 cP medium. Bile-duct phantom navigation used 10 Hz and a maximum field of 125 mT; fresh porcine bile-duct navigation used 10 Hz.	Propulsion force/torque: NR. Peak speeds in the 1 cP medium were 5.25 and 9.57 mm s^−1^ at the two working points. Ex vivo navigation covered 3.5 cm in approximately 20 s, followed by module separation in approximately 5 s. Stent radial force was 0.516 N cm^−1^, not propulsion force/torque.	3T3 co-culture for 5 d showed no significant viability difference from the control. The bile-duct study was ex vivo, and no in vivo magnetic navigation damage threshold was established.
Acoustic; bubble microrobot [90]	Penetration depth and formal spatial/temporal control resolution: NR. The robot was tested at 133 kHz and 7 Vpp in a PBS-filled PDMS chamber; hydrophone calibration was reported separately.	Propulsion force/torque: NR. Speed reached approximately 3000 µm s^−1^ at 133 kHz and 7 Vpp.	Non-active printed robots showed no damage in hMSC tests lasting up to 72 h. Active operation produced a cell-lysis endpoint; the article does not support a single consistent lysis percentage or a formal tissue-damage threshold.
Optical/photothermal; microrocket [92]	Tissue penetration depth and formal actuation resolution: NR. An 808 nm laser at 1.5 W drove motion in 50% glycerol; a specific steering response of approximately 152 degrees in 1.1 s was demonstrated.	Propulsion force/torque: NR. Mean speed was 2.8 mm s^−1^, approximately 62 body lengths s^−1^, in the viscosity-matched solution.	A biomedical compatibility assay and a measured tissue-damage threshold were not reported. The study identified the required actuation intensity and local heating as limitations for in vivo use.
Electric; ferroelectric nematic microrobot [93]	Penetration depth and formal spatial/temporal control resolution: NR. Motion was measured in an ITO electrode cell at 6 kHz and 120 V rms; in a separate cell, speed rose from approximately 250 to 450 µm s^−1^ as voltage increased from 100 to 104 V.	Force/torque: NR. Median speed was approximately 300 µm s^−1^. Activity lasting 10–20 min under one SU-8 condition is an operating lifetime, not temporal resolution.	Physiological compatibility and tissue-damage threshold: NR. The electrode-defined liquid crystal cell was not a biomedical implantation model.
Chemical/enzymatic; silica micromotor [94]	Penetration depth and spatial/temporal control resolution: NR. Urease-functionalized hollow silica micromotors were tested with urea concentrations up to 100 mM, using 20 micromotors per condition.	Force/torque: NR. Speed reached 2.07 +/− 0.25 µm s^−1^ at 100 mM urea; glucose oxidase- and aldolase-functionalized particles did not exceed passive controls under the reported conditions.	Physiological compatibility and tissue-damage threshold: NR. This was an aqueous mechanism study without cell, tissue, mucus, animal or in vivo safety testing.
Biohybrid; magnetic bacterial carrier [95]	Quantitative tissue penetration depth and formal spatial/temporal control resolution: NR. Fields of 5, 10, 15 and 20 mT were used for motility comparisons, 10 mT for square-path steering, and 26 mT in separate spheroid-guidance and collagen-invasion experiments.	Force/torque: NR. Average speed was 18.5 +/− 4.7 µm s^−1^ at 10 mT. Guidance toward HT-29 spheroids and collagen-gel invasion were separate task outputs; the latter did not report penetration depth.	The study was in vitro. Spheroid viability after the specified NIR treatment does not establish systemic biosafety, bacterial immune compatibility, long-term clearance or a tissue-damage threshold.

Note: NR indicates not reported in the cited study. Values are reproduced only from the cited representative studies. Tissue penetration, control resolution, propulsion force or torque, compatibility assays and damage endpoints are not interchangeable across robot sizes, media, input hardware or biological models.

**Table 2:** Source-verified quantitative comparison of representative imaging and readout studies.

Imaging/Readout and Representative Study	Depth, Detection and Spatial Performance	Temporal Performance and Drive Compatibility	Model and Evidence Boundary
HFUS/photoacoustic imaging [47]	In vivo penetration depth and formal detection limit: NR. At 800 nm, in-plane axial resolution was 75 µm; single approximately 100 µm micromotors were tracked beneath 5 and 10 mm of ex vivo chicken tissue.	Acquisition: 5–20 fps. External magnetic steering was jointly demonstrated with HFUS/photoacoustic imaging.	Phantom, ex vivo chicken tissue and in vivo mouse bladder/uterus studies. The 5 and 10 mm values are ex vivo tissue thicknesses; object size is not a formal detection limit, and frame rate is not closed-loop command rate.
Volumetric MSOT [38]	In vivo penetration depth and formal detection limit: NR. Signal was detectable beneath approximately 7 cm of ex vivo soft brain tissue; LOT distinguished vascular structures separated by 22 µm over an approximately 1.5 mm cortical depth range.	In vivo volumetric acquisition: 100 Hz. A rotating NdFeB electromagnet was operated during in vivo brain imaging.	Ex vivo porcine tissue and in vivo mouse tail, leg and brain vasculature. The 7 cm value is ex vivo; 22 µm is vascular structure separation rather than robot localization error; 100 Hz is acquisition, not closed-loop rate.
Color-flow ultrasound [129]	In vivo penetration depth and formal detection limit: NR. Five 60–80 µm microrobots were detected at approximately 10 cm in an agar phantom with a 4.0–16.0 MHz ultrasound scanner.	Frame rate: NR. Color-flow imaging and approximately 100 kHz acoustic actuation were demonstrated simultaneously.	Agar phantom and ex vivo mouse bladder. The 10 cm value is a phantom distance; 60–90 µm is demonstrated object size, not a formal detection limit; 4.0–16.0 MHz is imaging frequency, not frame rate.
Magnetic particle imaging [48]	In vivo penetration depth and formal detection limit: NR. Images of two TriMag robots began to merge at a measured separation of approximately 0.243 mm; 3D MPI localization and CT-MPI tracking were demonstrated.	Temporal rate: NR. Magnetic actuation and MPI were used in staged workflows; simultaneous actuation during MPI acquisition was not specified.	Agar/Petri-dish tests, tissue-filled vessel phantom, ex vivo porcine eye and in vivo mouse stomach. The 0.243 mm value is a system-specific merging boundary, not universal MPI resolution.
X-ray guidance [115]	In vivo penetration depth and formal detection limit: NR. Contrast-dependent visibility windows were 65, 35 and 25 s for 570, 420 and 330 µm robots; phantom guidance used 100 kV and 100 µA.	Frame rate: NR. Real-time X-ray guidance and magnetic steering were used together.	Branching-vessel phantom and in vivo rat-liver targeting. Visibility duration is not temporal resolution, robot diameter is not a formal detection limit, and the X-ray settings are phantom-specific.
MRI; postoperative readout [115]	In vivo penetration depth and formal detection limit: NR. MNP relaxivities were r1 = 0.53 mM^−1^ s^−1^ and r2 = 174.13 mM^−1^ s^−1^; groups of 100 robots with diameters of 330, 420 or 570 µm were visible in T2-weighted phantom images.	Temporal rate: NR. MRI was performed after magnetic targeting; the field gradient was near zero during acquisition, so drive and imaging were staged rather than simultaneous.	MRI phantom and postoperative in vivo rat-liver distribution. The 330 µm value is robot diameter in a 100-robot group, not a single-robot detection limit; relaxivity is not spatial resolution.
NIR-IIb fluorescence [130]	Quantitative in vivo penetration depth and formal detection limit: NR. In a QD-filled capillary under 1% Intralipid, SBR fell from approximately 50 at 0 mm to approximately 1.4 at 10–11 mm; image FWHM rose from 51.1 to 70.9 µm.	Imaging reached 50 fps with 3–10 ms exposure; selected in vivo experiments used approximately 16.6 fps. External NdFeB magnetic navigation was jointly demonstrated.	Capillary/intralipid depth test and in vivo mouse vascular, peritoneal and gastrointestinal studies. The 10–11 mm value is not in vivo depth; FWHM is capillary-image width, not localization error or a formal detection limit.
Optical coherence tomography [131]	In vivo penetration depth, formal detection limit and measured spatial resolution: NR. Spectral-domain OCT recorded a 6.5 µm pixel size.	A 76 kHz acquisition/image-speed setting was reported. Tracking was demonstrated during visible-light actuation; magnetic/acoustic drive compatibility was not tested.	Aqueous or vitreous-humor samples and ex vivo enucleated porcine eyes. The 76 kHz setting is not 76,000 fps, 6.5 µm is pixel size rather than measured resolution, and the eye study was ex vivo.

Note: NR indicates not reported in the cited study. Phantom or ex vivo depth is not treated as in vivo penetration; demonstrated object size or separability is not treated as a formal detection limit; acquisition rate is not treated as closed-loop command rate; and simultaneous and staged drive-imaging workflows are distinguished.

## Data Availability

No new data were created or analyzed in this study. Data sharing is not applicable to this article.
